# Characterization of cognitive decline in long-duration type 1 diabetes by cognitive, neuroimaging, and pathological examinations

**DOI:** 10.1172/jci.insight.180226

**Published:** 2025-01-30

**Authors:** Hetal S. Shah, Matthew N. DeSalvo, Anastasia Haidar, Surya Vishva Teja Jangolla, Marc Gregory Yu, Rebecca S. Roque, Amanda Hayes, John Gauthier, Nolan Ziemniak, Elizabeth Viebranz, I-Hsien Wu, Kyoungmin Park, Ward Fickweiler, Tanvi J. Chokshi, Tashrif Billah, Lipeng Ning, Atif Adam, Jennifer K. Sun, Lloyd Paul Aiello, Yogesh Rathi, Mel B. Feany, George L. King

**Affiliations:** 1 Dianne Hoppes Nunnally Laboratory Research Division, Joslin Diabetes Center, Boston, Massachusetts, USA.; 2 Department of Medicine, Beth Israel Deaconess Medical Center, Harvard Medical School, Boston, Massachusetts, USA.; 3 Department of Radiology, and; 4 Psychiatry Neuroimaging Laboratory, Brigham and Women’s Hospital, Boston, Massachusetts, USA.; 5 Beetham Eye Institute, Joslin Diabetes Center, Boston, Massachusetts, USA.; 6 Department of Ophthalmology, Harvard Medical School, Boston, Massachusetts, USA.; 7 Department of Pathology, Brigham and Women’s Hospital, Boston, Massachusetts, USA.

**Keywords:** Aging, Endocrinology, Dementia, Diabetes

## Abstract

**BACKGROUND:**

We aimed to characterize factors associated with the under-studied complication of cognitive decline in aging people with long-duration type 1 diabetes (T1D).

**METHODS:**

Joslin “Medalists” (n = 222; T1D ≥ 50 years) underwent cognitive testing. Medalists (n = 52) and age-matched nondiabetic controls (n = 20) underwent neuro- and retinal imaging. Brain pathology (n = 26) was examined. Relationships among clinical, cognitive, and neuroimaging parameters were evaluated.

**RESULTS:**

Compared with controls, Medalists had worse psychomotor function and recall, which associated with female sex, lower visual acuity, reduced physical activity, longer diabetes duration, and higher inflammatory cytokines. On neuroimaging, compared with controls, Medalists had significantly lower total and regional brain volumes, equivalent to 9 years of accelerated aging, but small vessel disease markers did not differ. Reduced brain volumes associated with female sex, reduced psychomotor function, worse visual acuity, longer diabetes duration, and higher inflammation, but not with glycemic control. Worse cognitive function, lower brain volumes, and diabetic retinopathy correlated with thinning of the outer retinal nuclear layer. Worse baseline visual acuity associated with declining psychomotor function in longitudinal analysis. Brain volume mediated the association between visual acuity and psychomotor function by 57%. Brain pathologies showed decreased volumes, but predominantly mild vascular or Alzheimer’s-related pathology.

**CONCLUSION:**

To our knowledge, this is the first comprehensive study of cognitive function, neuroimaging, and pathology in aging T1D individuals demonstrated that cognitive decline was related to parenchymal rather than neurovascular abnormalities, unlike type 2 diabetes, suggestive of accelerated aging in T1D. Improving visual acuity could perhaps be an important preventive measure against cognitive decline in people with T1D.

**FUNDING:**

The Beatson Foundation, NIH/NIDDK grants 3P30DK036836-34S1 and P30DK036836-37, and Mary Iacocca fellowships.

## Abstract 2

This first comprehensive study of cognitive function, brain imaging and pathology in aging T1D individuals indicates that cognitive decline in T1D is different from T2D, and improving visual acuity could be a novel preventive measure.

## Introduction

Both type 1 (T1D) and type 2 (T2D) diabetes are rapidly increasing in prevalence globally and affect more than 33 million people in the United States, including 25% of the aging population (people >65 years) (1). Along with other major complications, cognitive dysfunction is now recognized as a major morbidity associated with diabetes, and poses great socioeconomic burdens and reduced quality of life (2–5). Diabetes-associated cognitive decline may have multiple underlying mechanisms, including vascular abnormalities and changes in glucose, insulin sensitivity, and amyloid metabolism (2, 3). Studies examining diabetes-associated cognitive dysfunction have mostly focused on T2D, which itself, along with insulin resistance and obesity, are significant risk factors for dementia especially in Alzheimer’s disease and related disorders (6). Detailed clinical characterization of cognitive dysfunction in a large cohort with long T1D duration has been limited given that living longer than 55 years among those with T1D has only become possible recently (7, 8). Studies in younger T1D populations have suggested the important roles of poor glycemic control and hypoglycemia in cognitive decline, but their role in older T1D populations remains undefined (2, 3, 9, 10). Additionally, modifiable risk factors for T1D-associated cognitive decline may differ in older age groups compared with younger people (11). Brain imaging studies in T1D have been restricted to younger to mid-life adults, with reports of vascular and parenchymal changes (12). However, it is not clear whether these changes can be extrapolated to older individuals with long-standing T1D (12). No studies have comprehensively characterized both neuroimaging and pathological brain examinations and their relationships to cognitive changes in T1D. Furthermore, as retinal neurodegeneration is an early event in the pathogenesis of diabetic retinopathy and could run in parallel with brain neurodegeneration (13–17), it is important to further tease out these relationships in the context of T1D.

The Joslin 50-year Medalist Study, a cohort of individuals (“Medalists”) with 50 or more years of T1D, is exceptionally well suited to characterize and identify potential novel markers of cognitive decline, especially since they do not exhibit clinical signs of insulin resistance and have relatively good glycemic and metabolic control (18–23). Yet, as reported, Medalists had similar levels of cognitive impairment to those of age-matched individuals with T2D and greater levels of impairment compared with age-matched individuals without diabetes (24). Thus, we aimed to characterize factors associated with cognitive decline in this elderly cohort with very long duration of T1D in a first comprehensive study of cognitive and clinical assessments, retinal and brain imaging, and gross and histopathological examination of postmortem brain specimens.

## Results

### Baseline characteristics of study groups.

Supplemental Table 1 (supplemental material available online with this article; https://doi.org/10.1172/jci.insight.180226DS14) summarizes the baseline characteristics of Medalists in the overall cohort (*n* = 1034), and the following subsets: cognitive (*n* = 222), brain imaging (*n* = 52), longitudinal (*n* = 48), and brain donors (*n* = 26). At baseline, Medalists were 55% female, mean age 66 years, with 53 years of T1D, mean glycated hemoglobin (HbA1c) of 7.2%, and body mass index (BMI) of 26 kg/m^2^. While 77% of Medalists reported a history of hypertension, the average systolic and diastolic blood pressures were very well controlled, coupled with a cardiac-favorable lipid profile (mean LDL cholesterol [LDL-C] of 81 mg/dL and HDL-C of 65 mg/dL) and mean estimated glomerular filtration rate (eGFR) of 69.7 mL/min/1.73 m^2^, normal for the age group. Approximately 80% of Medalists reported that they do exercise. A third of Medalists had proliferative diabetic retinopathy (PDR), 40% had reported cardiovascular disease (CVD), but only 12% had diabetic nephropathy (DN). Participants of the various subsets were fairly representative of the overall study population, except for the brain imaging group which had a smaller proportion of individuals with CVD (19% vs. 40%) and DN (2% vs. 13%) at baseline. Only 17% had at least one APOE risk allele as compared with 26% in the overall study. The brain donor group had a higher proportion of individuals with CVD (46%) and DN (15%) at baseline than the overall Medalist cohort (Supplemental Table 1).

Cross-sectional characteristics of the study participants at time of cognitive or neuroimaging study visit, or last visit for brain donors, are shown in Table 1. Mean age at the time of the cognitive study was 71 years and duration of diabetes 61 years. Other characteristics are comparable to the baseline study visit. In the neuroimaging study group, there were approximately 50% females and an average age at study visit of 72 years, with higher HbA1c as expected, although still very good glycemic control, and a better lipid profile compared with age-matched nondiabetic controls (Table 1). At the time of brain MRI, the proportion of those with self-reported hypertension was 72% for Medalists and 47% for controls, while CVD was reported for 35% of the Medalists as compared with only 22% among control individuals (Table 1). There were no significant differences between Medalists and controls with respect to sex, age, BMI (both were in the non-overweight range), renal function, education, or lifestyle.

### Cognitive function.

**Figure 1:**
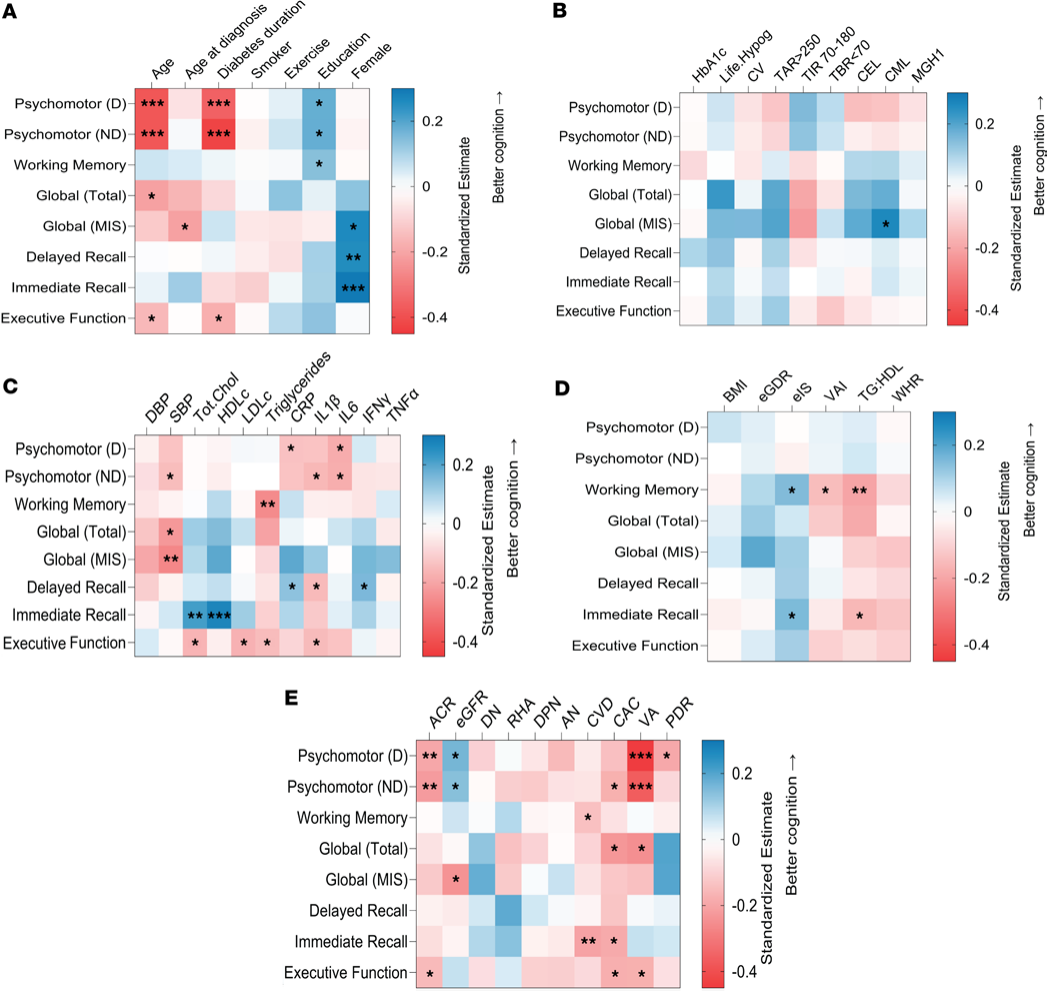
Associations of clinical characteristics with cognitive function in T1D (n = 222). Heatmaps showing bivariate standardized estimates of associations between clinical characters as independent variables and cognitive function domains as dependent variables in linear regression models. The estimates represent changes in cognitive function per 1 SD change in the clinical predictor. Color and intensity represent the strength and direction (positive, zero or no, and negative) of association. Better cognitive function is represented by more blue estimates, and worse by red. MIS, memory index score. Blank squares not significant. *P < 0.05, **P < 0.01, ***P < 0.0001. (**A**) Markers of sociodemographics and lifestyle. (**B**) Glycemic markers. Life.Hypog, lifetime hypoglycemia severity; CV, coefficient of variation of glucose on CGM; TAR>250, time above range of glucose > 250 mg/dL; TIR 70–180, time-in-range 70–180 mg/dL; TBR<70, time below range of glucose < 70 mg/dL; CEL, CML, and MGH1 are advanced glycation end products. (**C**) Cardiometabolic markers. DBP and SBP, diastolic and systolic blood pressure; CRP, C-reactive protein; IL, interleukin; IFN-γ, interferon γ; TNF-α, tumor necrosis factor α. (**D**) Insulin resistance markers. BMI, body mass index; eGDR, estimated glucose disposal rate; eIS, estimated insulin sensitivity; VAI, visceral adiposity index; TG:HDL, triglyceride/HDL ratio; WHR, waist/hip ratio. (**E**) Complications. ACR, urine albumin/creatinine ratio; eGFR, estimated glomerular filtration rate; D and ND, dominant and nondominant hands; DN, diabetic nephropathy; RHA, reduced hypoglycemia awareness; DPN, diabetic peripheral neuropathy; AN, autonomic neuropathy; CVD, cardiovascular disease; CAC, coronary artery calcification; VA, visual acuity; PDR, proliferative diabetic retinopathy.

Compared with controls, Medalists had significantly worse recall, psychomotor function, and global cognition (Table 1). In bivariate analysis among Medalists, worse psychomotor function in both dominant and nondominant domains was (*P* < 0.05) associated with increased age, longer duration of diabetes, increased interleukin 6 (IL-6), worse renal function (higher albumin/creatinine ratio [ACR] and lower eGFR), and lower visual acuity. Better function was associated with higher education (Figure 1). Worse motor function in the dominant hand also associated significantly with higher C-reactive protein (CRP) and PDR. In the nondominant hand it was associated with higher systolic blood pressure, IL-1β, and coronary artery calcification (CAC) (Figure 1). In multivariable models incorporating all these significant covariates, only education, duration, and visual acuity remained significant (*P* < 0.05) for both dominant and nondominant hands, while IL-6 remained significant for the dominant hand (Supplemental Table 2).

Worse immediate recall was associated (*P* < 0.05) with higher triglyceride/HDL ratio, CVD, and CAC. Better immediate recall was associated with female sex, higher HDL, and total cholesterol, and better insulin sensitivity (Figure 1). Better delayed recall (*P* < 0.05) was associated with female sex, CRP, and interferon **γ** (IFN-γ) levels, while worse delayed recall was associated with IL-1β (Figure 1). In multivariable models, female sex remained significant for both types of recall, while IL-1β and IFN-**γ** also remained significant for delayed recall (Supplemental Table 2). Worse executive function associated significantly with higher age, duration, LDL, ACR, triglycerides, total cholesterol, IL-1β, CAC, and with lower visual acuity (Figure 1). In multivariable models, total cholesterol and IL-1β remained significant for associations with executive function (Supplemental Table 2). Better working memory was associated significantly with higher education and estimated insulin sensitivity index (eIS), while worse working memory was associated with higher triglycerides, triglyceride/HDL ratio, visceral adiposity index (VAI), and CVD (Figure 1). However, none of these remained significant in multivariable analysis (Supplemental Table 2). None of the cognitive domains were associated with HbA1c, lifetime hypoglycemia severity, continuous glucose monitoring (CGM) indices, or advanced glycation end products (AGEs), including carboxymethyl-lysine (CML), carboxyethyl-lysine (CEL), and methylglyoxal-hydroimidazolone-1 (MGH1) (Figure 1B).

Better global cognition delayed recall (captured by memory index score [MIS]) was associated with female sex and CML, while worse outcome was associated with age at diagnosis, SBP, and eGFR. Worse total global cognition was associated with increasing age, SBP, and CAC, and with reduced visual acuity. However, in multivariable models, female sex, SBP, and eGFR remained significant for the global cognition recall, while visual acuity remained significant for the total global cognition (Supplemental Table 2).

For physical activity reported in the Paffenbarger surveys, in age-, sex-, and education-adjusted models, increased numbers of daily stairs climbed and blocks walked were associated with better executive function and psychomotor speed, respectively (*P* < 0.05) (Supplemental Table 3). Increased weekly duration of, and caloric expenditure from, swimming associated with better delayed recall (Supplemental Table 3).

From the lifestyle activity questionnaire (LAQ), driving was associated with better executive function (Supplemental Table 3). Sewing and talking about politics reduced psychomotor function, while going to the movies was associated with poorer executive function (Supplemental Table 3).

Higher adherence to Mediterranean, Dietary Approaches to Stop Hypertension (DASH) and Empirical Index for Hyperinsulinemia (EDIH) diets were associated with better working memory (Supplemental Table 4). The Mediterranean-DASH Intervention for Neurodegenerative Delay (MIND) diet was not significantly associated with any of the cognitive domains. Higher antiinflammatory score for the Empirical Dietary Inflammatory Pattern (EDIP) index was associated with worse executive function (Supplemental Table 4).

### Structural volumetrics.

**Figure 2:**
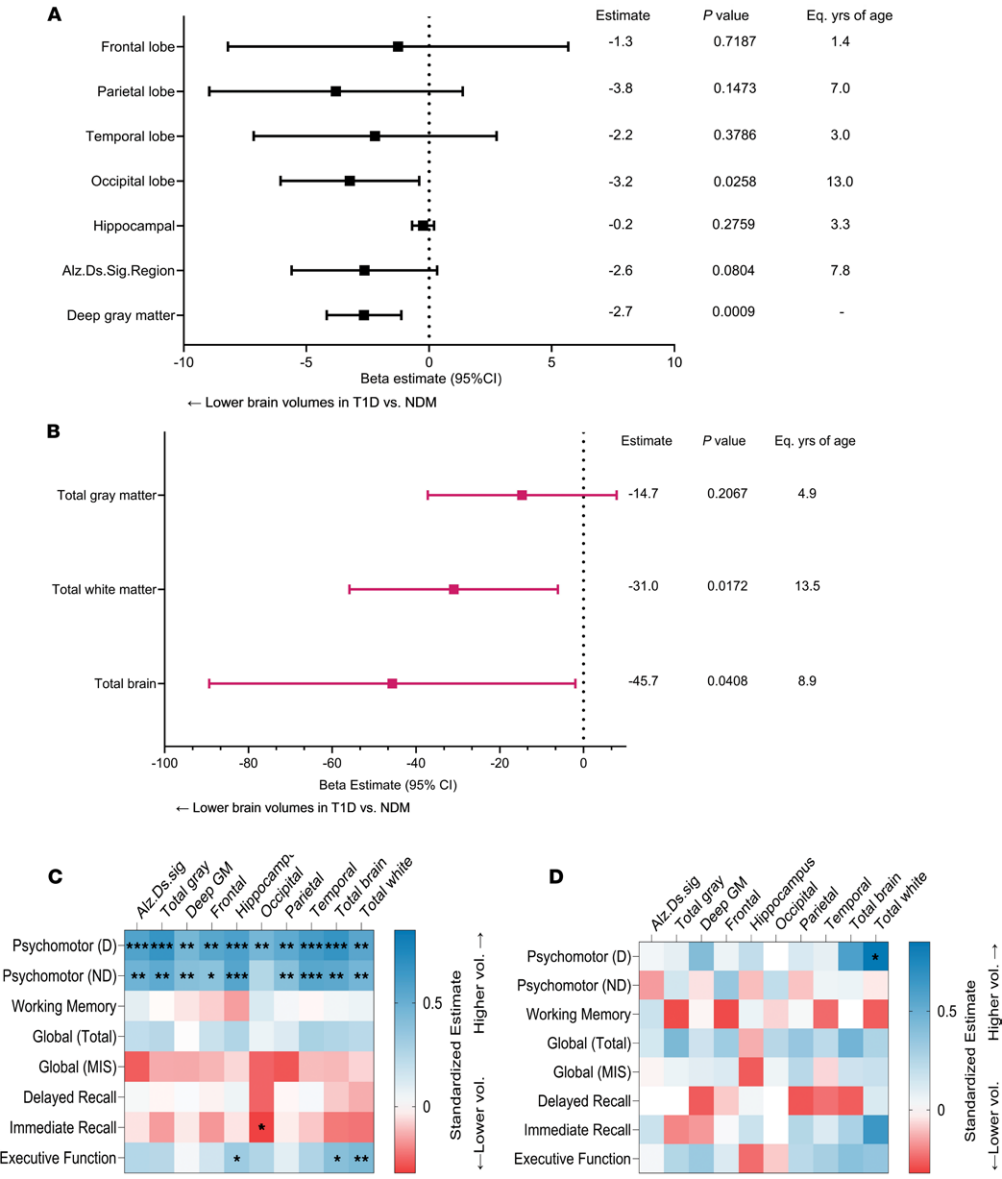
Brain structure and cognitive function in Medalists (T1D, n = 52) versus nondiabetic controls (NDM, n = 20). (**A** and **B**) Differences in brain volumes between T1D and NDM. Forest plots showing β estimates and 95% confidence intervals from linear regression associations between regional (**A**) and total (**B**) brain volumes and case (T1D) versus control (NDM) status, adjusted by intracranial volumes. Eq.yrs of age, equivalent years of aging of T1D brain compared to NDM; Alz.Ds.Sig., Alzheimer’s disease signature region. (**C** and **D**) Associations between brain volumes and cognitive function. Heatmaps showing bivariate standardized estimates of associations between brain volumes and cognitive function in T1D (**C**) and NDM controls from linear regression models (**D**). Better cognitive function is represented by more blue estimates, and worse cognitive function by red. D and ND, dominant and nondominant hands; MIS, memory index score; Deep GM, deep gray matter. Blank squares not significant. *P < 0.05, **P < 0.01, ***P < 0.0001.

Compared with nondiabetic individuals, Medalists had significantly (*P* < 0.05) lower volumes in regions of the total brain, total gray matter, total white matter, occipital lobe, and deep gray matter — which is the subcortical region comprising the basal ganglia and the thalamus; and a marked but nonsignificant (*P* = 0.08) lower volume in the Alzheimer’s disease signature region, composed of hippocampal, parahippocampal, entorhinal, and related areas (Figure 2, A and B). These volume reductions translated to Medalists’ brains being approximately 8–13 years older than those of nondiabetic individuals (Figure 2, A and B).

In models adjusted for intracranial volume, lower volumes across all brain regions examined in the T1D Medalists were associated with worse motor function (Figure 2C). Nominal associations were observed between better executive function and higher total brain, total white matter, and hippocampal volumes, while immediate recall associated with occipital lobe volume (Figure 2C). In models additionally adjusted for age, sex, and education, these associations remained significant (*P* < 0.05), except the following: motor function (both dominant and nondominant) with total white matter and frontal lobe volumes, immediate recall and motor function (dominant) with occipital lobe volume, motor (nondominant) with frontal lobe volume, and motor (dominant) with deep gray matter volume (Supplemental Table 5).

In control individuals, none of the above associations between cognitive function and brain volumes were observed, except motor function of the dominant side, which was associated with reduced total white matter volume (Figure 2D).

**Figure 3:**
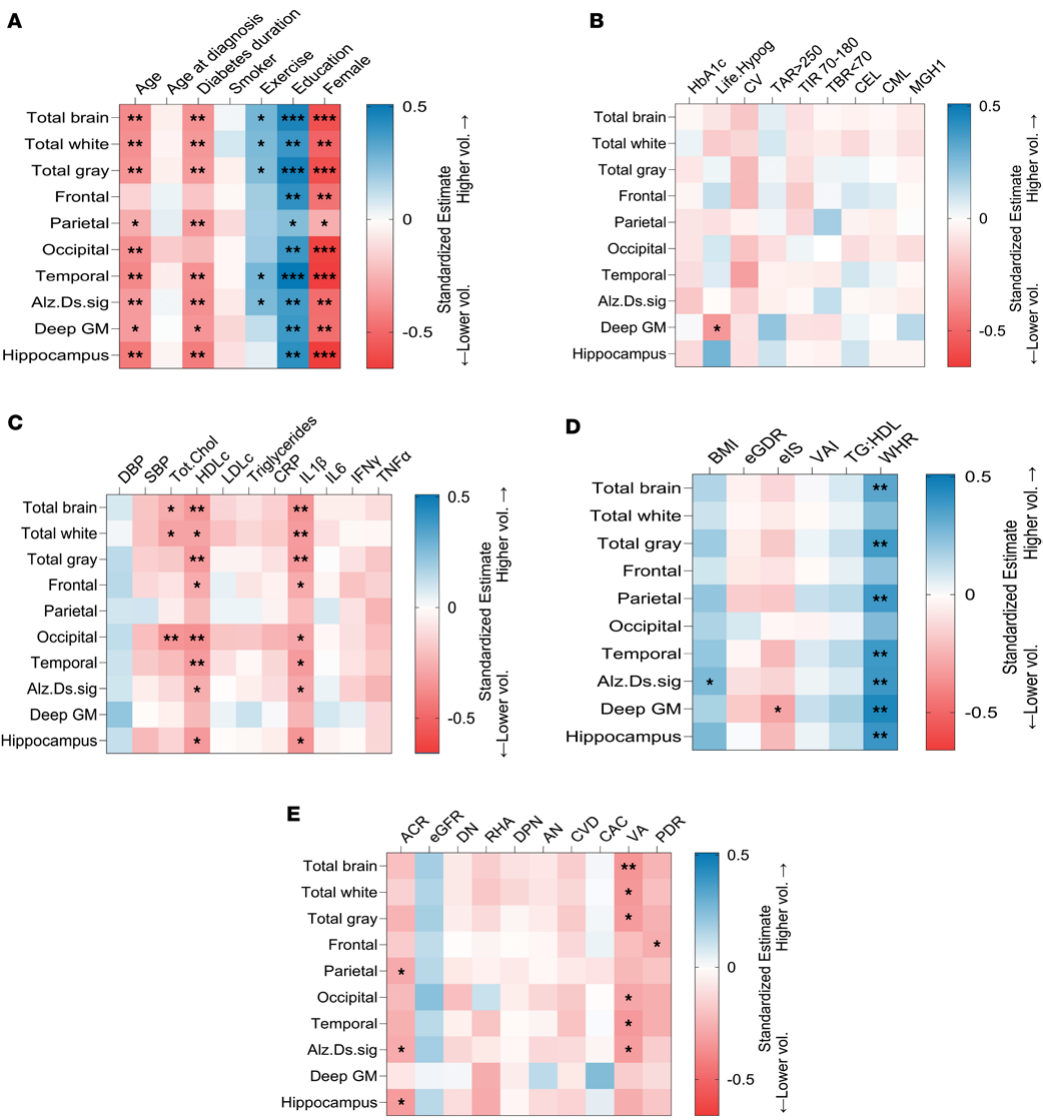
Associations of clinical characteristics with brain volumes in T1D (*n* = 52). Heatmaps showing bivariate standardized estimates of associations between clinical characters as independent variables and brain volumes as dependent variables from linear regression models. The estimates represent changes in brain volume per 1 SD change in the clinical predictor. Color and intensity represent the strength and direction (positive, zero or no, and negative) of association. Higher brain volume is represented by more blue estimates, and lower by red. Blank squares not significant. *P < 0.05, **P < 0.01, ***P < 0.0001. (**A**) Markers of sociodemographics and lifestyle. (**B**) Glycemic markers. Life.Hypog, lifetime hypoglycemia severity; CV, coefficient of variation of glucose on CGM; TAR>250, time above range of glucose > 250 mg/dL; TIR 70–180, time-in-range 70–180 mg/dL; TBR<70, time below range of glucose <70 mg/dL; CEL, CML, and MGH1 are advanced glycation end products. (**C**) Cardiometabolic markers. DBP and SBP, diastolic and systolic blood pressure; CRP, C-reactive protein; IL, interleukin; IFN-γ, interferon γ; TNF-α, tumor necrosis factor α. (**D**) Insulin resistance markers. BMI, body mass index; eGDR, estimated glucose disposal rate; eIS, estimated insulin sensitivity; VAI, visceral adiposity index; TG:HDL, triglyceride/HDL ratio; WHR,waist/hip ratio. (**E**) Complications. ACR, urine albumin/creatinine ratio; eGFR,estimated glomerular filtration rate; DN, diabetic nephropathy; RHA, reduced hypoglycemia awareness; DPN, diabetic peripheral neuropathy; AN, autonomic neuropathy; CVD, cardiovascular disease; CAC, coronary artery calcification; VA, visual acuity; PDR, proliferative diabetic retinopathy.

In Medalists, education, exercise, and waist/hip ratio associated with higher volumes in most regions, while female sex, increasing age, diabetes duration, HDL, total cholesterol, IL-1β, and lower visual acuity associated with lower brain volumes (Figure 3). A higher BMI and lower TNF-α also associated with a higher volume of the Alzheimer’s disease signature region. PDR was associated with decreased frontal lobe volume (Figure 3). Lifetime hypoglycemic severity was associated with lower deep gray matter volume. In multivariable models accounting for all significant associations for each region, sex remained significant for all volumes except parietal lobe (Supplemental Table 6). Additionally, visual acuity remained significant for associations with Alzheimer’s disease signature region, temporal lobe, total brain, and total white and gray matter volumes. IL-1β remained significant for associations with total brain, total white and gray volumes, hippocampal, and Alzheimer’s disease signature regions (Supplemental Table 6). Duration remained significant for total brain, total white matter, and Alzheimer’s disease signature region. For the latter region, HDL and TNF-α also remained significant. Education also remained significant for frontal lobe volume, age remained significant for occipital lobe volume, and lifetime hypoglycemic severity for deep gray matter region. For parietal lobe volume, only ACR remained significant (Supplemental Table 6).

There were no associations between brain volumes and age at diagnosis, HbA1c, CGM indices, or AGEs (Figure 3B). Lifetime hypoglycemic severity was associated (*P* < 0.05) with lower volume of the deep gray matter region, but not with other volumes (Figure 3B). Other markers of inflammation or insulin resistance did not correlate with volumes (Figure 3, C and D). Except for PDR, no associations were observed with diabetic vascular complications (Figure 3E).

### Vascular imaging.

**Figure 4:**
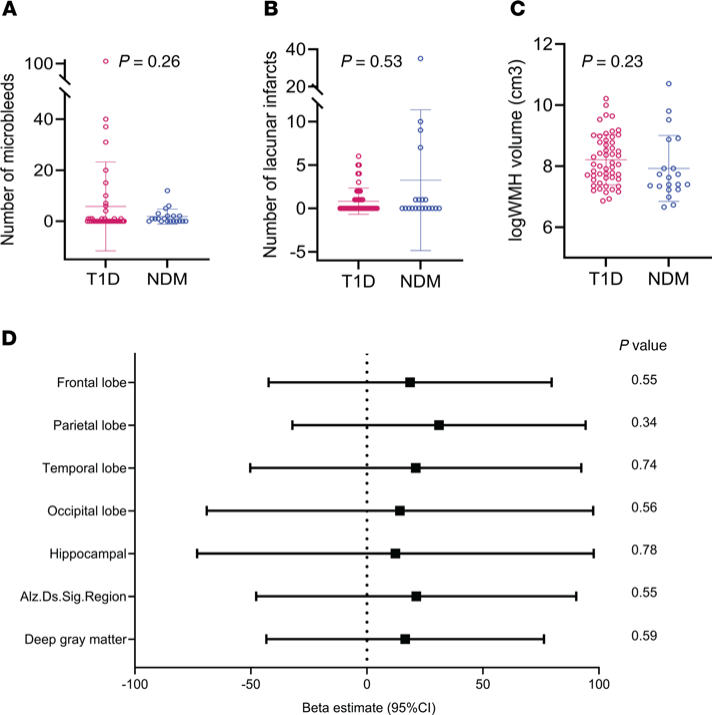
Vascular imaging in Medalists (T1D, *n* = 52) versus nondiabetic (NDM, *n* = 20) controls. Differences between T1D and NDM for counts of microbleeds (**A**) and lacunar infarcts (**B**), volume of white matter hyperintensities (WMHs) (**C**) and regional cerebral perfusion (**D**). Dot plots show mean ±SD. Mann-Whitney *U* tests were used to test for significant differences in microbleeds and lacunar infarcts. WMH volumes were log-normalized and then differences were examined by Student’s *t* test. Linear regression models tested for relationship between cerebral perfusion and case-control status; β estimates and 95% confidence intervals are shown in the forest plot.

No significant differences were observed between Medalists and controls in the number of microbleeds, volume of white matter hyperintensities (WMHs), or number of lacunar infarcts (Figure 4, A–C), nor were any differences observed in cerebral perfusion rates (Figure 4D).

WMHs did not associate with cognitive function in the Medalists (Supplemental Figure 1A). Better motor function of the nondominant side was associated with higher occipital lobe perfusion, while worse global cognition associated with higher perfusion of the deep gray matter region (Supplemental Figure 2A). In control individuals, higher cerebral perfusion in all regions was associated with worse executive function (Supplemental Figure 2B).

Increased WMHs were associated with worse renal function and higher coronary artery calcification scores (Supplemental Figure 1, B and C). Cerebral perfusion improved with female sex, higher HDL, HbA1c, ACR, and insulin sensitivity, and with lower BMI, DBP, waist/hip ratio, LDL, cholesterol, and CRP (Supplemental Figure 2C). In multivariable models, mainly BMI and sex remained significant. ACR remained significant for temporal lobe and hippocampus. LDL was significant for hippocampus and Alzheimer’s disease signature region. CRP, in addition to age and sex, remained associated with deep gray matter perfusion (Supplemental Figure 2C). There were no associations seen between WMHs or cerebral perfusion with other complications (CVD, PDR, DN, neuropathy), markers of insulin resistance, vascular markers, hypoglycemia or CGM parameters, or AGEs.

### Histopathology.

**Figure 5:**
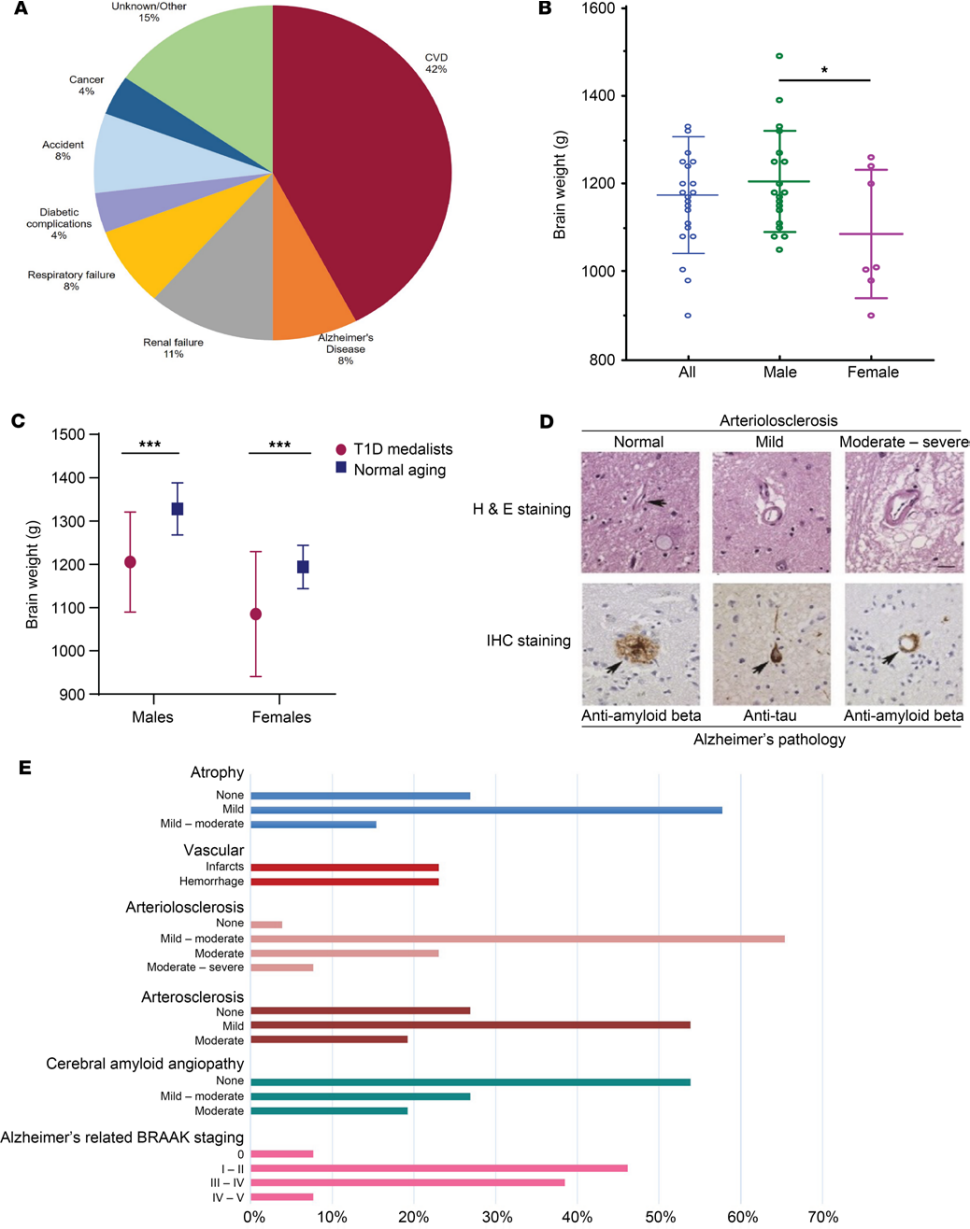
Gross and histopathological exams of 26 brains from T1D Medalists. (**A**) Causes of death among 26 brain donors. CVD, cardiovascular disease. (**B**) Distribution of brain weights in Medalists. Data are presented as mean ± SD. (**C**) Comparison of mean (±SD) brain weights for T1D Medalists (*n* = 19 males and 7 females) compared to summary data from normal referenced aging population (*n* = 546 males and 465 females). *P < 0.05, ***P < 0.0001 from standardized *t* tests. (**D**) Arteriolosclerosis and Alzheimer’s pathology. Hematoxylin and eosin (H&E) staining of normal vessel with thin wall (upper left, arrow), mild arteriolosclerosis with thickening of vessel wall and widened perivascular space (upper middle), and moderate-to-severe arteriolosclerosis with thickened vessel wall and surrounding parenchymal damage (upper right). Scale bar: 50 μm. Immunohistochemistry (IHC) staining of amyloid plaque (lower left, arrow), neurofibrillary tangle (lower middle, arrow), and vessel involved by cerebral amyloid angiopathy (lower right, arrow). (**E**) Summary of brain gross and histopathological exams (*n* = 26).

Characteristics of brain donors at their last visit are shown in Table 1, and summaries of their brain histopathology are shown in Figure 5, Table 2, and Supplemental Table 7. Approximately 42% of the brain donors died due to CVD, 11% due to renal failure, and 8% due to Alzheimer’s disease (Figure 5A). The average brain weight was 1173 ± 133 g, and females’ brains (*n* = 7) were on average 120.3 ± 54.5 g lighter than male brains (*P* = 0.037) (Figure 5B). Medalists’ average brain weights for both males and females were significantly (*P* < 0.0001) lower than those in comparative age groups (66+ years) in a referenced normal aging population (25) (Figure 5C). Brain volumes were mildly reduced (Figure 5D). Vascular pathology (infarcts, bleeding, and atherosclerosis) was mostly mild (Figure 5, D and E) as per standard guidelines (26, 27). As expected in this older age group, there was evidence of Alzheimer’s-related deposits, as per Braak staging (28), such that 8% had no deposits, 46% had Braak stage I-II involvement, 38% had stage III-IV involvement, 8% had stage IV-V involvement (Figure 5, D and E).

For 3 of the 5 brain donors who had previous cognitive assessments, cognitive scores deviated by 1.5 SD of the means of those without diabetes. Despite poor cognitive scores, 2 of these individuals had only Braak stage I Alzheimer’s pathology, and 1 had Braak III-IV along with neocortical Lewy body pathology (Table 2). Of the 2 patients who had been diagnosed with Alzheimer’s disease, 1 had Braak stage I-II and the other had stage V (Table 2). The patient that had 2 APOE risk alleles had Braak stage IV pathology (Table 2).

### Retinal imaging.

**Figure 6:**
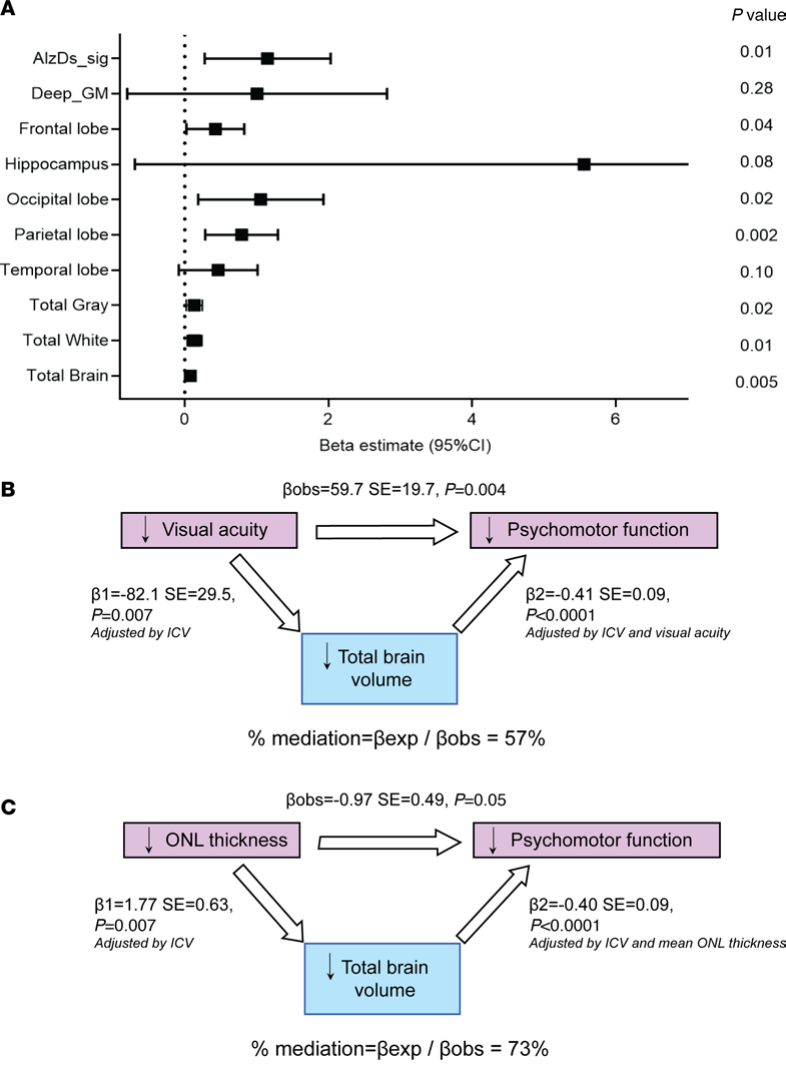
Relationships between outer nuclear layer (ONL) thickness, visual acuity, and brain volumes in T1D (*n* = 52). (**A**) ONL thickness and brain volumes in T1D. Linear regression β estimates and 95% confidence intervals shown in forest plot. Alz.Ds.Sig., Alzheimer’s disease signature region; Deep_GM, deep gray matter. (**B** and **C**) Mediation analysis. Estimating how much of the association between visual acuity (**B**) or ONL thickness (**C**) and brain volume is mediated by psychomotor function. Each arrow represents a linear regression model with the dependent variable at the arrowhead and the independent variable at the arrow base. βobs, observed β; βexp, expected β; SE, standard error of the mean.

Since visual acuity and PDR consistently correlated with various parameters of cognitive dysfunction and brain volumetric losses, we evaluated detailed changes in retinal layers in relation to these brain parameters. Increased thickness of the retinal outer nuclear layer (ONL), composed primarily of photoreceptors, was significantly associated with higher brain volumes in all regions except the deep gray matter, hippocampus, and temporal lobe (Figure 6A). Other retinal layers did not associate with brain volumes (Supplemental Figure 3). Mediation analysis showed that total brain volume mediated 57% of the association between visual acuity and psychomotor function (Figure 6B), and 73% of the association between ONL thickness and psychomotor function (Figure 6C).

### Longitudinal study.

A subset of Medalists (*n* = 48) had 2 or more cognitive visits with an average follow-up time of 4.8 years between visits (Table 1 and Supplemental Table 1). Declines were observed in recall and psychomotor cognitive domains (Supplemental Table 8), although none were significant. A strong association was observed between lower visual acuity at first cognitive visit and reduced psychomotor function in the nondominant side over time, independent of age, sex, or duration of T1D (Supplemental Table 8).

## Discussion

In this first comprehensive study to our knowledge of clinical and cognitive testing, retinal and neuroimaging, and brain gross and histopathology exams, we show that aging individuals with long-duration T1D, despite excellent glycemic and cardiometabolic profiles, have worse cognitive function compared with people without diabetes, and that this is related mainly to brain parenchymal loss and less to vascular or Alzheimer’s-related phenomena, unlike findings in T2D (29, 30).

Cognitive studies in aging populations with T1D have been sparse. Several studies, including ours, have reported cognitive decline in domains affecting recall and psychomotor function (7, 24, 31, 32). Lack of associations with hypoglycemia and cognitive function or brain structure in our study are consistent with previous reports in prospective studies, including the Diabetes Control and Complications Trial (DCCT) and the Stockholm Diabetes Intervention Study (33, 34). However, unlike previous reports (9, 31, 34), our study did not observe an association between hyperglycemia and cognitive function. Perhaps this lack of association between HbA1c and cognitive function or brain structure may be due to Medalists’ tight glycemic control and little variability in HbA1c. This is unlikely though, as other short- and long-term measures of glycemic control, including CGM metrics or AGEs, in our study did not associate significantly with cognitive decline. Moreover, intensive glycemic control in the DCCT and Action to Control Cardiovascular Risk in Diabetes (ACCORD) trial have not shown benefit for cognitive function (35, 36). Nonetheless, despite long duration of excellent glycemic, lipid, and blood pressure control, Medalists still exhibit significant cognitive decline and lower brain volumes compared with people without diabetes, pointing to yet uncovered T1D-related factors that may play a role in their cognitive decline.

The results of brain imaging and pathological exams in this study differ from those observed in individuals with T2D. While global atrophy is also a feature commonly seen in T2D, the volume reduction is generally modest, comparable to 3–5 years of normal aging (12, 29), while we are observing 9 years of accelerated global atrophy in T1D compared with normal aging individuals. Furthermore, regional volume loss in T2D predominantly affects memory areas like the hippocampal regions or temporal lobe (37), while we observed atrophy predominantly in the occipital lobe and subcortical regions of the basal ganglia and thalamus. Additionally, small vessel disease has been commonly and consistently reported in T2D, including WMHs and lacunar infarcts on MRI and histopathology (29, 30), whereas our findings did not show significant increases in small vessel disease in T1D Medalists compared to nondiabetic controls, which were confirmed by minimal vascular pathology in postmortem brain examinations.

In T2D, while more than 40% of people have moderate-to-severe Alzheimer’s-related pathology in brain autopsies, multiple etiologies have been suggested for cognitive decline, including vascular and cerebral insulin resistance, inflammation, or endothelial dysfunction due to accumulation of AGEs or toxic lipids or proteins in the vasculature (38–41). For the Medalists with chronic T1D, Alzheimer’s-related immunostaining, including amyloid plaques and Braak staging of tau, showed predominantly mild-to-moderate pathology, even in the 3 individuals who had poor cognitive function, the one individual with 2 APOE risk alleles, and one of the patients whose reported cause of death was Alzheimer’s disease. In the other patient who had a clinical Alzheimer’s diagnosis, Braak V staging was seen, with frequent amyloid plaques. One of the patients with poor cognitive function also had cortical Lewy body pathology along with Braak stage III-IV, suggestive of a mixed picture underlying their cognitive decline. Medalists also exhibited very well-controlled cardiometabolic profiles and insulin resistance markers were not associated with cognitive or neuroimaging outcomes, again suggesting that underlying mechanisms and pathologies of cognitive decline in T1D may differ from T2D. However, some inflammatory cytokines including IL-1β and IL-6 did associate with cognitive decline and lower brain volumes in our study, suggesting that the role of inflammation needs to be investigated.

Recently, neuroimaging studies by the DCCT and the Epidemiology of Diabetes Interventions and Complications (EDIC) in participants with a mean age of 59 years, and on average 38 years of T1D (31, 42) reported reductions in total brain, white and gray matter volumes, the latter being associated with poor cognitive function, while they reported no markers of Alzheimer’s-related neurodegeneration (31). However, their reports lacked pathological confirmation (42). Our study is the first to our knowledge to examine an aging population with mean age of 72 years and 50 or more years of T1D, where postmortem brain exams provided the strongest evidence alongside MRI changes that underlying pathologies of cognitive decline in aging individuals with long-duration T1D are probably unrelated to vascular or Alzheimer’s dementia, differing from T2D (29, 30).

Despite a low prevalence of nephropathy and excellent glycemic control without dyslipidemia, obesity, or insulin resistance, Medalists still exhibited significant cognitive decline with corresponding brain atrophy. Thus, it is important to identify potential factors that may mediate these effects. Female sex significantly associated with better cognitive function but lower brain volumes, consistent with previous reports in the general population and in people with diabetes where women perform better in cognitive tests than men (43–45), yet have a greater risk of dementia (46–48). Perhaps this is because women live longer than men, or perhaps their higher cognitive reserves during testing mask underlying functional aberrations, and thus they only get diagnosed at more advanced stages (45, 46). Our study supports this hypothesis, given the lower brain volumes observed in women despite better cognitive function. Female hormones have been suggested to play a protective role in brain health, but our study population includes mostly menopausal women given the age of the participants.

Diet and physical activity are known protective factors against cognitive decline (49), and our study supports their protective roles even in an elderly population with long-duration T1D and good cardiometabolic control. Specifically, the Mediterranean and DASH diets, with high contents of fresh fruits and vegetables and low amounts of processed meats, seem to have beneficial effects for working memory. Increased physical activity like number of stairs climbed or blocks walked daily were associated with better executive and psychomotor function, respectively, while swimming benefitted delayed recall.

An interesting finding in this study that has not been discussed in any of the cognitive studies involving T1D or T2D populations was the association of worse visual acuity with both worse cognitive function and lower brain volumes even after multivariable adjustments. These findings support our previous report showing associations between retinal ONL thickness and psychomotor function as well as severity of PDR (13). Indeed, the retina is a “window” to the brain due to shared embryonic origins, and as retinal neurodegeneration is an early event in the pathogenesis of DR, these could run parallel with brain neurodegeneration (13–17). While it is not surprising that loss of visual acuity will adversely affect psychomotor function during clinical testing, the significant association of both visual acuity and selective ONL thinning with brain volumes strongly indicated that there are biological interactions among these factors. Severity of PDR correlated with almost all retinal layers, yet psychomotor function and immediate recall were particularly affected by thinning of the ONL, which is composed mainly of photoreceptors, rods and cones (13). In addition, our results showed that total brain volume mediated 57% and 73% of the associations between psychomotor function and visual acuity or ONL thickness, respectively. Since severity of PDR is associated with changes in many retinal layers, the selective finding of only ONL thinning being associated with worse visual acuity and lower brain volumes supports the novel concept that visual acuity may have an independent effect on T1D-related cognitive decline. This is unlike Alzheimer’s and related disorders, where all retinal fiber layers and even retinal blood flow are affected (50). While preclinical studies in animals suggest that there is photoreceptor death in diabetes, this, however, has not been widely reported in human studies and warrants more investigations (51). It is possible that the reduction in mental stimulation resulting from poor visual acuity results in worse psychomotor function and its associated reduction of certain total and regional brain volumes (the sensory loss consequence theory) (52). This is supported by the longitudinal substudy, where visual impairment assessed at time of first cognitive visit was associated with further psychomotor function decline after 3–5 years of follow-up. Another possibility is that both visual impairment and cognitive decline have a common underlying mechanism, such as inflammation or neurodegeneration (53). As long-term exposure to hyperglycemia can lead to neuroinflammation in both the brain and retina, cognitive decline may be attributed to this common process rather than to the sensory loss theory. Nonetheless, it is also possible that this latter effect could contribute to worsening the progression of cognitive decline. Associations between visual acuity and cognitive functioning have been suggested in nondiabetic populations such as the Salisbury Eye Evaluation Study (54) and the Age-Related Eye Disease Study (55), among others (52, 56), but have remained undiscussed for diabetes. Thus, our findings suggest the interesting possibility that rigorously targeting improvements in visual acuity may be a crucial approach to primary and secondary prevention of cognitive decline in aging populations with T1D. The optimal timing and required nature of these interactions remains to be determined.

Our study has several strengths, including access to a well-characterized and richly phenotyped elderly population with long-duration T1D; detailed and high-quality cognitive, retinal, and neuroimaging assessments; all study participants including cases and controls underwent neuroimaging on the same MRI machine under the same protocol; availability of brain specimens and high-quality gross and histopathological examinations; and the opportunity to examine multiple modifiable risk factors beyond poor cardiometabolic profiles that are known to associate with cognitive decline.

Our study has certain limitations. This was a cross-sectional study with limited power and a small group of nondiabetic controls. However, we still observed relatively strong associations with our main findings and the additional longitudinal component lends a directionality to our hypothesis. Second, all of the 26 brain donors, except 5, did not have cognitive or brain MRI data to make direct correlations. However, the 4 individuals who had poor cognitive function and brain pathology only exhibited minimal Alzheimer’s and vascular pathology, thus supporting the MRI findings. Third, we were unable to make any inferences about mild cognitive impairment (MCI) or dementia-related outcomes, as the majority of the Medalists were cognitively intact. Perhaps Medalists are experiencing subtle diabetes-related cognitive decrements greater than controls but these are not impairing them functionally. Fourth, we did not assess more definitive Alzheimer’s-related biochemical or imaging markers, including PET scans or collection of cerebrospinal fluid for amyloid or tau assessment. Last, as the Medalists are a unique group of individuals with T1D having good cardiometabolic control, the lack of neurovascular contributions to their cognitive decline may not necessarily be generalizable to other populations with T1D and studies need to be conducted in aging non-Medalists. Future studies would also need to include a spectrum of cognitive functioning and imaging studies, including PET in a large-scale longitudinal follow-up research design, as well as brain pathological studies.

In conclusion, this is the first comprehensive study to our knowledge of cognitive function, brain imaging, and pathology in people with long-duration T1D and excellent cardiometabolic profiles. We have provided strong evidence that cognitive decline in T1D is associated with lower brain volumes, but not vascular or Alzheimer’s-related pathologies. In addition, besides changes in diet and activity levels, targeting visual acuity improvements and retinopathy severity may be crucial to preventing cognitive impairment in these individuals.

## Methods

## Sex as a biological variable

This study recruited both males and females in equal proportions. Sex as a biological variable is considered for all analyses.

## Study population

The Joslin 50-year Medalist Study is a well-characterized cohort of individuals (*n* = 1,033) recruited from across the United States with well-documented T1D for 50 or more years, as previously described (18–23). The study population consists of 50% females, 94% non-Hispanic Whites, 1.6% American Indian or Alaska Native, 0.45% non-Hispanic Blacks, 0.9% Hispanic, and 3% unreported ethnicity. They have been actively followed up and richly phenotyped at the Joslin Diabetes Center via comprehensive clinical exams, food frequency and exercise questionnaires, ophthalmic, renal, and cardiac exams, including imaging, general hematological and clinical chemistry studies, and mixed-meal tolerance studies. All patients undergo HLA typing and autoimmune markers, HbA1c, CRP, lipid and renal profiling, and C-peptide measurements. The bio-bank contains plasma, serum, and mononuclear cells from all participants, and postmortem organs (eyes, kidneys, pancreas, hearts, and brains) from a subset (18–23).

Thus far, 222 Medalists have undergone clinical cognitive testing, as well, 52 of these Medalist participants and 20 age-matched controls underwent additional brain imaging studies. The study was open to all 50-year Medalist Study participants who previously indicated willingness to be contacted regarding participation in further studies. Participants were included in the brain imaging studies if they did not have contraindications to MRI. A subset of Medalists (*n* = 48) who had at least 2 cognitive study visits formed part of the longitudinal substudy. Brain donors (*n* = 26) were Medalists who had previously consented for organ donation and from whom brains were procured for pathological exams. Of these brain donors, 5 individuals had previous cognitive testing. A flowchart of the study design is outlined in Supplemental Figure 4.

*Comprehensive clinical assessment*. Clinical and cognitive assessments done in the Medalist study have been described previously (13, 18–24). Briefly, the following measures were administered by trained staff at the study visit.

*General*. Updated medical history, complications, vitals, cardiometabolic assessments, and clinical labs were as per standard protocols by Quest Diagnostics.

*Cognition*. The cognitive battery included the following reliable, previously described measures (57): The Montreal Cognitive Assessment (MOCA, for global cognition) (58); The Wechsler Abbreviated Scale of Intelligence III (IQ); The Wechsler Memory Scale III (working memory); The Rey Auditory Verbal Learning Test (RAVLT, for immediate and delayed recall); The Delis-Kaplan Executive Function System (executive function); and The Grooved Pegboard (psychomotor speed and efficiency) (57). Standardized *t* scores were calculated for the memory domains, with increasing scores representing better cognition. Higher scores for motor domains represented worse times to complete the pegboard test. Participants were screened for possible MCI based on the following criteria: a cutoff value of less than 26 out of 30 points on the MOCA or less than 35 points on the RAVLT immediate or delayed recall tests (59).

*APOE4 genotypes*. APOE4 genotypes were extracted from the existing Medalists’ genomic database using tools such as PLINK 1.9 and GTOOL.

*Physical activity and lifestyle*. Physical activity was captured by Paffenbarger questionnaires (60), while lifestyle was via LAQs (61). Diet was captured using validated food frequency questionnaires (62, 63), and adherence to the following dietary patterns were derived as previously described, including alternate Mediterranean (aMed) (64), DASH (65), MIND (66, 67), EDIH, and EDIP (68).

### Cardiac assessments

CVD was captured by self-reported occurrence of coronary artery disease, myocardial infarction, angina, cardiac or leg angioplasty, or coronary bypass graft surgery. CAC CT scans were obtained using a 320 detector-row system (Aquilion ONE, Toshiba Medical Systems) and scored using prospective ECG gating without contrast. Agatston scores were derived by radiologists at Brigham and Women’s Hospital.

### Renal assessments

Serum creatinine and urine ACRs were measured via standard methods at Quest Diagnostics. Serum creatinine-based eGFR was calculated by the Chronic Kidney Disease Epidemiology Collaboration algorithm (69), and DN was defined using a cutoff eGFR of less than 45 mL/min/1.73 m^2^.

### Markers of insulin resistance

The waist/hip ratio was calculated with waist circumference measured at the midpoint between the iliac crest and the lower rib margin, while hip circumference was measured at the maximum circumference around the buttocks posteriorly and pubic symphysis anteriorly. Surrogate measures of insulin resistance were also evaluated using the estimated glucose disposal rate (eGDR) and eIS equations previously validated in adult T1D patients by the Pittsburgh Epidemiology of Diabetes Complications (EDC) (70) and Coronary Artery Calcification in Type 1 Diabetes (CACTI) studies (71), respectively. Other surrogate measures of insulin resistance included the previously validated visceral adiposity index (VAI) and the triglyceride/HDL ratio calculated from the standard lipid profile using values in units of mg/dL (72).

### Inflammatory markers

Serum CRP was measured by particle-enhanced immunonephelometry (BN ProSpec analyzer; Dade Behring). Inflammatory cytokine (IFN-γ, TNF-α, IL-6, IL-1β) concentrations were measured in human plasma using the Meso Scientific Discovery Multiplex electrochemiluminescence assay as per the protocol of the Proinflammatory Panel 1 (human) V-PLEX kit (catalog K151A9H) and the plasma samples were diluted 2-fold.

### CGM, hypoglycemia, and neuropathy assessments

Among existing CGM users, a single consecutive 14-day block of CGM data was remotely obtained after a period of performing usual activities in a subset of the cognitive study participants (*n* = 96). CGM indices of variability (CV), hyperglycemia (time-above-range, TAR > 250 mg/dL), or hypoglycemia (time-below-range TBR < 70 mg/dL) were extracted. The COMPASS-31 questionnaire (73) assessed hypoglycemia severity and frequency and autonomic neuropathy, and the Clarke Hypoglycemia Awareness Survey (74) assessed hypoglycemia awareness. Peripheral neuropathy was adjudicated by a Michigan Screening Instrument (MNSI) survey score of greater than 2 (75, 76).

### AGEs

CML, CEL, and MGH1 were measured using high-performance liquid chromatography–mass spectrometry adapted from previously described methods (77).

### Brain imaging

Scanning was performed on a Siemens 3 Tesla Prism scanner at the Brigham and Women’s Hospital imaging core as per the Alzheimer’s Disease Neuroimaging Initiative 3 (ADNI) advanced protocol guidelines (Supplemental Table 9) (78). For patient comfort and flexibility to adjust for kyphosis that is more prevalent in older cohorts, the 32 channel GE head coil was used. Structural volumetrics were acquired via the T1-weighted sequences and processed using Freesurfer image analysis suite (79). Regions of interest (ROIs) were derived by summing volumes of right and left hemispheres of various cortical and subcortical regions as described in Supplemental Table 10. Volumes of WMHs were extracted from the freesurfer output. Microbleeds and lacunar infarcts were manually counted in each individual brain scan by a board-certified neuroradiologist using the T2*GRE and 3D-FLAIR sequences. Arterial spin labeling (ASL) sequences assessed cerebral blood flow changes.

### Ocular assessments

These were performed at the Joslin Beetham Eye Institute, as previously described (13). Visual acuity was assessed using standardized Early Treatment Diabetic Retinopathy Study (ETDRS) chart (80). PDR was adjudicated at ETDRS score greater than 53 on 7 standard field fundus photos (81). Individual retinal layer thicknesses in the foveal 1 × 1 mm area were measured by spectral-domain optical coherence tomography–based (OCT-based) high-resolution scans with automated layer segmentation software (SPECTRALIS v6.0; Heidelberg Engineering). The following layers were evaluated: retinal nerve fiber (RNFL), ganglion cell (GCL), inner and outer plexiform (IPL and OPL), inner and outer nuclear (INL and ONL), and retinal pigment epithelium (RPE) (82).

### Brain gross pathology and histopathology

Over 400 Medalists have consented to donate organs postmortem. Brains were procured from 26 Medalist donors by a trained retrieval team from the National Disease Research Interchange (NDRI). Fresh brains were examined for any grossly evident pathology (atrophy, focal and vascular lesions), and then sagittally hemisected and formalin fixed. A standard histopathological paraffin-embedded blocking procedure and hematoxylin/eosin staining was applied. Selected sections were immunostained with antibodies directed toward β-amyloid, tau, and α-synuclein. Interpretation of vascular and neurodegenerative pathology followed standard guidelines (26, 27). Alzheimer’s-related pathology was graded as per Braak staging (28). Under the supervision of our consultant neuropathologist, brain sectioning and histopathology were done at Brigham and Women’s Hospital.

For comparison of Medalists’ brain weights with those of a normal aging population, summary level data were obtained from a previous study compiling brain weights from autopsy reports of 2,773 males and 1,963 females, who were all White individuals across the lifespan, from hospitals in Washington DC and Maryland (25). For our study comparison, mean (SD) brain weights of individuals aged 66 or older were extracted for 546 men and 465 women (25).

### Statistics

All analyses were conducted in SAS v9.4. Descriptive statistics are reported as means (±SD) or frequencies (*n*, %) as appropriate. Differences between T1D Medalists and controls were examined via unpaired Student’s *t* tests for continuous variables, and Pearson’s χ^2^ tests for independence applied to categorical variables. For non–normally distributed variables, data were log-transformed as appropriate or the Mann-Whitney *U* test, a nonparametric alternative to the *t* test, was used. Cross-sectional relationships of cognitive or imaging parameters with clinical characteristics were assessed using generalized linear regression models. Significant variables (*P* < 0.05) from the bivariate analyses were selected as potential confounders in multivariable linear regression models testing relationships between cognitive and brain imaging parameters in T1D Medalists. Generalized linear regression models adjusting for intracranial volume (ICV) were applied to examine differences in brain imaging parameters between Medalists and controls. The equivalent years of age for volume differences between Medalists and controls were obtained from linear regression models by calculating the ratio of the β estimate for caseness to that for age when both variables and ICV were included as independent variables and MRI volumes were the dependent variables.

For illustration purposes, we used standardized estimates (where the estimates represent changes in the dependent variable per 1 SD change in the independent variable) for the relationship between various clinical characters and cognitive functions or brain volumes. Then, we used these estimates and respective *P* values to plot heatmaps where color and intensity represent the strength and direction (positive, zero or no, and negative) of association.

To evaluate the relationships between thickness of retinal nerve layers (derived by OCT) and cognitive or brain imaging parameters, we used generalized estimating equations with an unstructured correlation matrix accounting for within-subject correlations, as data from both eyes of each individual were included in the model.

To assess the mediating effects of brain volume on the relationship between visual acuity or ONL thickness and psychomotor function, a mediation analysis was performed. The observed β estimate (βobs) was derived from the linear regression model testing the effects of visual acuity on psychomotor function. The linear regression effect estimate (β1) of visual acuity (or ONL) on total brain volume (adjusted by ICV) was multiplied by the estimate (β2) derived from the linear regression between total brain volume and psychomotor function (with ICV and visual acuity as covariates), resulting in the expected β (βexp). The βexp was divided by the observed (βexp/βobs) to derive the percentage mediation.

For the longitudinal analysis, paired *t* tests were used to examine differences between cognitive function at first visit compared to follow-up visit. Change in cognitive function was derived by subtracting the follow-up visit scores from the first visit scores for each domain, and linear regression models were applied to test the relationships between visual acuity from the first visit and change in cognitive function.

### Study approval

The study followed written informed consent procedures, was conducted in accordance with the guidelines provided by the Declaration of Helsinki, and was overseen by the Joslin Committee on Human Studies institutional review board.

### Data availability

All datasets underlying the main and supplemental tables and figures have been made available in the supplemental Supporting Data Values file.

## Author contributions

HSS designed and conducted the study, acquired and analyzed data, and wrote the manuscript. MND, AH, SVTJ, RSR, AH, TB, LN, AA, YR, LPA, and MBF analyzed data and reviewed the manuscript. MGY, JG, NZ, EV, IHW, KP, WF, TJC, and JKS acquired data and reviewed the manuscript. GLK designed the study, acquired data, and wrote the manuscript.

## Supplementary Material

Supplemental data

ICMJE disclosure forms

Supporting data values

## Figures and Tables

**Table 2:**
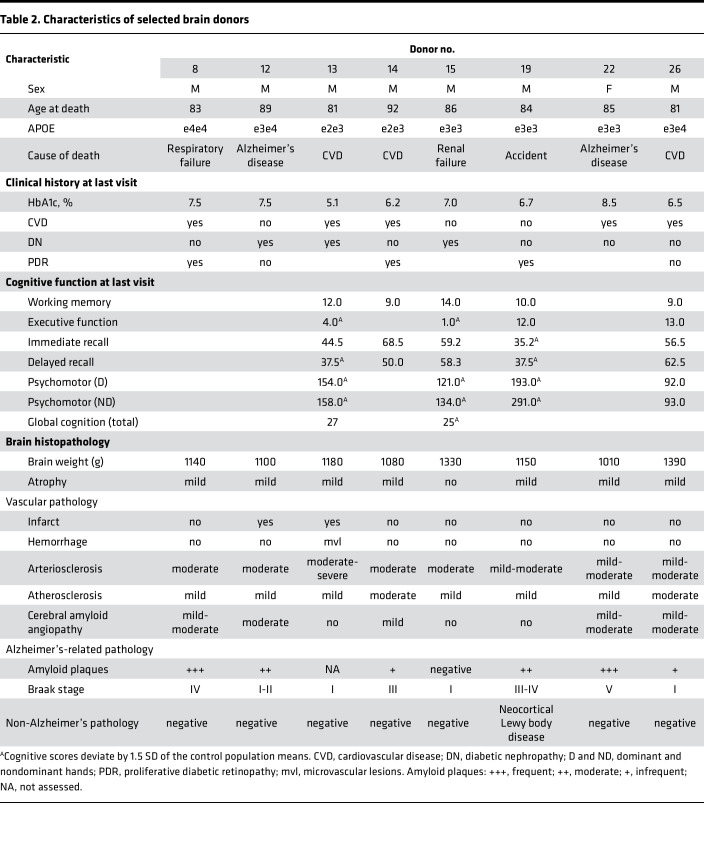
Characteristics of selected brain donors

**Table 1:**
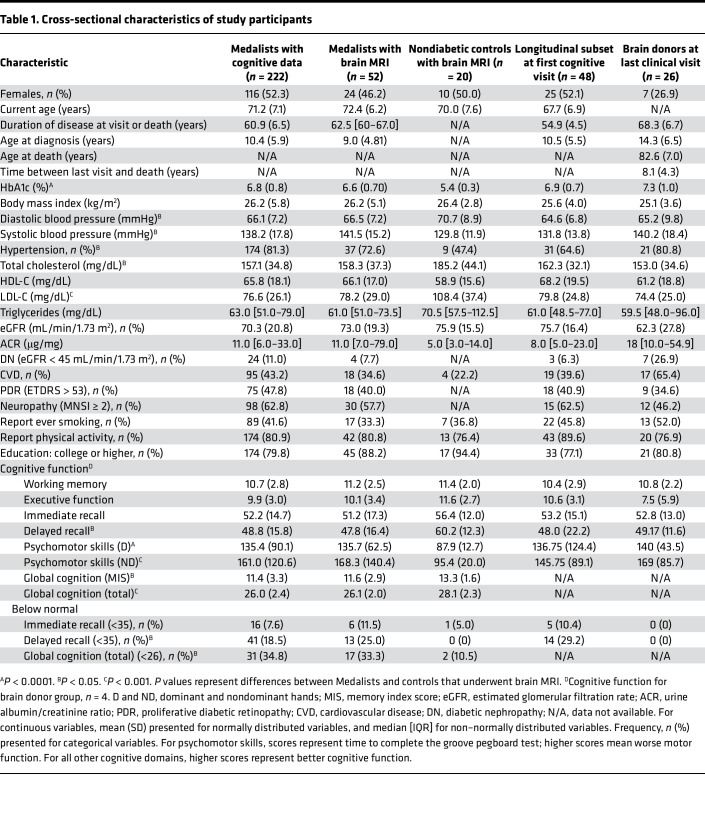
Cross-sectional characteristics of study participants

## Version 1 01/30/2025

In-Press Preview

## Version 2 03/10/2025

Electronic publication

## References

[1] https://www.cdc.gov/diabetes/php/data-research/index.html.

[2] Biessels GJ (2006). Risk of dementia in diabetes mellitus: a systematic review. Lancet Neurol.

[3] Gudala K (2013). Diabetes mellitus and risk of dementia: a meta-analysis of prospective observational studies. J Diabetes Investig.

[4] American Diabetes Association (2013). Economic costs of diabetes in the U.S. in 2012. Diabetes Care.

[5] Munshi MN (2017). Cognitive dysfunction in older adults with diabetes: what a clinician needs to know. Diabetes Care.

[6] Kuo CL (2018). Population-based cohort study on dementia risk in patients with type 1 diabetes mellitus. Neuroepidemiology.

[7] Brands AM (2005). The effects of type 1 diabetes on cognitive performance: a meta-analysis. Diabetes Care.

[8] Krolewski AS (1996). Epidemiology of late diabetic complications. A basis for the development and evaluation of preventive programs. Endocrinol Metab Clin North Am.

[9] Lacy ME (2018). Long-term glycemic control and dementia risk in type 1 diabetes. Diabetes Care.

[10] Lacy ME (2020). Severe hypoglycemia and cognitive function in older adults with type 1 diabetes: the study of longevity in diabetes (SOLID). Diabetes Care.

[11] Moheet A (2015). Impact of diabetes on cognitive function and brain structure. Ann N Y Acad Sci.

[12] Biessels GJ, Reijmer YD (2014). Brain changes underlying cognitive dysfunction in diabetes: what can we learn from MRI?. Diabetes.

[13] Fickweiler W (2021). Association of cognitive function and retinal neural and vascular structure in type 1 diabetes. J Clin Endocrinol Metab.

[14] Simo R (2018). Neurodegeneration in diabetic retinopathy: does it really matter?. Diabetologia.

[15] Pedersen FN (2023). Structural and metabolic retinal changes associated with mild cognitive impairment in type 2 diabetes. Diabetes.

[16] Little K (2022). Common pathways in dementia and diabetic retinopathy: understanding the mechanisms of diabetes-related cognitive decline. Trends Endocrinol Metab.

[17] Vujosevic S (2020). Screening for diabetic retinopathy: new perspectives and challenges. Lancet Diabetes Endocrinol.

[18] Gordin D (2019). Characterization of glycolytic enzymes and pyruvate kinase M2 in type 1 and 2 diabetic nephropathy. Diabetes Care.

[19] Keenan HA (2007). Clinical factors associated with resistance to microvascular complications in diabetic patients of extreme disease duration: the 50-year medalist study. Diabetes Care.

[20] Keenan HA (2010). Residual insulin production and pancreatic β-cell turnover after 50 years of diabetes: Joslin Medalist study. Diabetes.

[21] Qi W (2017). Pyruvate kinase M2 activation may protect against the progression of diabetic glomerular pathology and mitochondrial dysfunction. Nat Med.

[22] Sun JK (2011). Protection from retinopathy and other complications in patients with type 1 diabetes of extreme duration: the joslin 50-year medalist study. Diabetes Care.

[23] Tinsley LJ (2017). Association of glycemic control with reduced risk for large-vessel disease after more than 50 years of type 1 diabetes. J Clin Endocrinol Metab.

[24] Musen G (2018). Cognitive function deficits associated with long-duration type 1 diabetes and vascular complications. Diabetes Care.

[25] Dekaban AS (1978). Changes in brain weights during the span of human life: relation of brain weights to body heights and body weights. Ann Neurol.

[26] Hyman BT (2012). National Institute on Aging-Alzheimer’s Association guidelines for the neuropathologic assessment of Alzheimer’s disease. Alzheimers Dement.

[27] McKeith IG (2005). Diagnosis and management of dementia with Lewy bodies: third report of the DLB Consortium. Neurology.

[28] Braak H, Braak E (1991). Neuropathological stageing of Alzheimer-related changes. Acta Neuropathol.

[29] Biessels GJ, Despa F (2018). Cognitive decline and dementia in diabetes mellitus: mechanisms and clinical implications. Nat Rev Endocrinol.

[30] Pruzin JJ (2018). Review: Relationship of type 2 diabetes to human brain pathology. Neuropathol Appl Neurobiol.

[31] Jacobson AM (2022). Brain structure among middle-aged and older adults with long-standing type 1 diabetes in the DCCT/EDIC study. Diabetes Care.

[32] Ryan CM (1997). Effects of diabetes mellitus on neuropsychological functioning: a lifespan perspective. Semin Clin Neuropsychiatry.

[33] Diabetes C (2007). Long-term effect of diabetes and its treatment on cognitive function. N Engl J Med.

[34] Reichard P (1996). Complications in IDDM are caused by elevated blood glucose level: the Stockholm diabetes intervention study (SDIS) at 10-year follow up. Diabetologia.

[35] [no authors listed] (1996). Effects of intensive diabetes therapy on neuropsychological function in adults in the Diabetes Control and Complications Trial. Ann Intern Med.

[36] Launer LJ (2011). Effects of intensive glucose lowering on brain structure and function in people with type 2 diabetes (ACCORD MIND): a randomised open-label substudy. Lancet Neurol.

[37] Savelieff MG (2022). Diabetes and dementia: clinical perspective, innovation, knowledge gaps. J Diabetes Complications.

[38] Biessels GJ, Reagan LP (2015). Hippocampal insulin resistance and cognitive dysfunction. Nat Rev Neurosci.

[39] Arnold SE (2018). Brain insulin resistance in type 2 diabetes and Alzheimer disease: concepts and conundrums. Nat Rev Neurol.

[40] Beckman JA, Creager MA (2016). Vascular complications of diabetes. Circ Res.

[41] Basta G (2004). Advanced glycation end products and vascular inflammation: implications for accelerated atherosclerosis in diabetes. Cardiovasc Res.

[42] Habes M (2023). Patterns of regional brain atrophy and brain aging in middle- and older-aged adults with type 1 diabetes. JAMA Netw Open.

[43] McArdle JJ (2007). Latent variable analyses of age trends of cognition in the Health and Retirement study, 1992-2004. Psychol Aging.

[44] Downer B (2015). A summary score for the Framingham Heart Study neuropsychological battery. J Aging Health.

[45] Moran C (2021). Sex, diabetes status and cognition: findings from the study of longevity in diabetes. BMJ Open Diabetes Res Care.

[46] Nebel RA (2018). Understanding the impact of sex and gender in Alzheimer’s disease: a call to action. Alzheimers Dement.

[47] [no authors listed] (2022). 2022 Alzheimer’s disease facts and figures. Alzheimers Dement.

[48] Chatterjee S (2016). Type 2 diabetes as a risk factor for dementia in women compared with men: a pooled analysis of 2.3 million people comprising more than 100,000 cases of dementia. Diabetes Care.

[49] Krivanek TJ (2021). Promoting successful cognitive aging: a ten-year update. J Alzheimers Dis.

[50] Mirzaei N (2020). Alzheimer’s retinopathy: seeing disease in the eyes. Front Neurosci.

[51] Kern TS, Berkowitz BA (2015). Photoreceptors in diabetic retinopathy. J Diabetes Investig.

[52] Lindenberger U, Baltes PB (1994). Sensory functioning and intelligence in old age: a strong connection. Psychol Aging.

[53] Gaire BP (2024). Alzheimer’s disease pathophysiology in the Retina. Prog Retin Eye Res.

[54] Zheng DD (2018). Longitudinal associations between visual impairment and cognitive functioning: the Salisbury Eye Evaluation study. JAMA Ophthalmol.

[55] Clemons TE (2006). Cognitive impairment in the Age-Related Eye Disease study: AREDS report no. 16. Arch Ophthalmol.

[56] Anstey KJ (2001). Two-year decline in vision but not hearing is associated with memory decline in very old adults in a population-based sample. Gerontology.

[58] Nasreddine ZS (2005). The Montreal cognitive assessment, MoCA: a brief screening tool for mild cognitive impairment. J Am Geriatr Soc.

[59] Damian AM (2011). The Montreal cognitive assessment and the mini-mental state examination as screening instruments for cognitive impairment: item analyses and threshold scores. Dement Geriatr Cogn Disord.

[60] (1978). Physical activity as an index of heart attack risk in college alumni. Am J Epidemiol.

[61] Carlson MC (2012). Lifestyle activities and memory: variety may be the spice of life. The women’s health and aging study II. J Int Neuropsychol Soc.

[62] Willett WC (1985). Reproducibility and validity of a semiquantitative food frequency questionnaire. Am J Epidemiol.

[63] Rimm EB (1992). Reproducibility and validity of an expanded self-administered semiquantitative food frequency questionnaire among male health professionals. Am J Epidemiol.

[64] Fung TT (2009). Mediterranean diet and incidence of and mortality from coronary heart disease and stroke in women. Circulation.

[65] Fung TT (2008). Adherence to a DASH-style diet and risk of coronary heart disease and stroke in women. Arch Intern Med.

[66] Morris MC (2015). MIND diet slows cognitive decline with aging. Alzheimers Dement.

[67] Morris MC (2015). MIND diet associated with reduced incidence of Alzheimer’s disease. Alzheimers Dement.

[68] Lee DH (2020). Dietary inflammatory and insulinemic potential and risk of type 2 diabetes: results from three prospective U.S. cohort studies. Diabetes Care.

[69] Inker LA (2012). Estimating glomerular filtration rate from serum creatinine and cystatin C. N Engl J Med.

[70] Williams KV (2000). Can clinical factors estimate insulin resistance in type 1 diabetes?. Diabetes.

[71] Duca LM (2016). Development and validation of a method to estimate insulin sensitivity in patients with and without type 1 diabetes. J Clin Endocrinol Metab.

[72] Amato MC (2010). Visceral Adiposity Index: a reliable indicator of visceral fat function associated with cardiometabolic risk. Diabetes Care.

[73] Sletten DM (2012). COMPASS 31: a refined and abbreviated composite autonomic symptom score. Mayo Clin Proc.

[74] Clarke WL (1995). Reduced awareness of hypoglycemia in adults with IDDM. A prospective study of hypoglycemic frequency and associated symptoms. Diabetes Care.

[75] Feldman EL (1994). A practical two-step quantitative clinical and electrophysiological assessment for the diagnosis and staging of diabetic neuropathy. Diabetes Care.

[76] Herman WH (2012). Use of the Michigan Neuropathy Screening Instrument as a measure of distal symmetrical peripheral neuropathy in type 1 diabetes: results from the Diabetes Control and Complications Trial/Epidemiology of Diabetes Interventions and Complications. Diabet Med.

[77] Beisswenger PJ (2014). Detection of diabetic nephropathy from advanced glycation endproducts (AGEs) differs in plasma and urine, and is dependent on the method of preparation. Amino Acids.

[78] Weiner MW (2017). The Alzheimer’s disease neuroimaging initiative 3: continued innovation for clinical trial improvement. Alzheimers Dement.

[79] Fischl B (2012). FreeSurfer. Neuroimage.

[80] Ferris FL (1996). Standardizing the measurement of visual acuity for clinical research studies: guidelines from the Eye Care Technology Forum. Ophthalmology.

[81] Aiello LP (2019). Comparison of early treatment diabetic retinopathy study standard 7-field imaging with ultrawide-field imaging for determining severity of diabetic retinopathy. JAMA Ophthalmol.

