# Supplementary material for: Characterization of cognitive decline in long-duration type 1 diabetes by cognitive, neuroimaging, and pathological examinations
Source: JCI Insight. 2025 Jan 30;10(5):e180226. doi: 10.1172/jci.insight.180226 (PMC11949075; doi:10.1172/jci.insight.180226)

Supplement to Shah et al. Characterization of cognitive decline in long-duration type 1 diabetes by cognitive, neuroimaging and pathological examinations

Supplementary Table 1: Baseline characteristics of the 50-year Medalist study

Baseline Characteristic	Overall (N=1034)	Cognitive Subset (N=222)	BMRI Subset (N=52)	Longitudinal Subset (N=48)	Brain donors (N=26)
Sex (female), N (%)	563 (54.5)	116 (52.3)	24 (46.2)	25 (52.1)	7 (26.9)
Age (years)	66.07 (7.68)	63.8 (6.4)	61. 94 (5.68)	63.0 (6.0)	70.9 (6.8)
Duration of disease (years)	53 [51-57]	52 [51-55]	52 [51-53.5]	52.5 (2.9)	56.6 (5.5)
Age at Diagnosis (years)	11.12 (6.38)	10.38 (5.87)	8.96 (4.81)	10.5 (5.5)	14.3 (6.5)
HbA1c (%)	7.20 (0.96)	7.06 (0.80)	7.14 (0.70)	7.0 (0.6)	7.48 (1.33)
Body mass index (kg/m^2)	26.18 (4.69)	25.88 (4.38)	25.66 (3.37)	25.8 (3.4)	25.2 (3.4)
Diastolic blood pressure (mmHg)	64.22 (8.57)	65.18 (8.01)	64.43 (8.65)	63.9 (7.8)	64.9 (9.6)
Systolic blood pressure (mmHg)	132.40 (16.93)	132.20 (17.00)	129.3 (16.3)	130.5 (15.7)	132.2 (20.1)
Hypertension, N (%)	765 (76.5)	150 (70.4)	31 (59.6)	26 (55.3)	20 (76.9)
Total cholesterol (mg/dL)	161.53 (33.31)	163.01 (32.22)	166.25 (29.90)	163.6 (26.7)	162.5 (38.6)
High-density lipoprotein cholesterol (mg/dL)	65.29 (19.83)	67.40 (19.33)	66.15 (18.67)	69.6 (16.9)	64.6 (21.9)
Low-density lipoprotein cholesterol (mg/dL)	81.08 (24.39)	80.91 (24.19)	86.61 (21.43)	80.9 (21.4)	81.8 (26.6)
Triglycerides (mg/dL)	65.0 [50.0-89.0]	64.0 [50.0-85.0]	58.0 [48.5-87.0]	61 [46.5-81.5]	59.5 [48.0-110.0]
eGFR (ml/min/1.73m^2)	69.7 (20.4)	75.6 (19.1)	81.9 (15.7)	76.9 (15.6)	65.4 (21.9)
ACR (ug/mg)	12.0 [6.6-32.7]	10.0 [5.1-20.0]	9.6 [6.8-16.6]	9.0 [5.1-13.0]	9.85 [7.2-37]
Diabetic nephropathy (eGFR <45 ml/min/1.73m^2), N (%)	131 (12.78)	17 (7.7)	1 (1.9)	2 (4.2)	4 (15.4)
Proliferative diabetic retinopathy (ETDRS>53), N (%)	428 (46.47)	84 (41.4)	16 (35.6)	17 (37.0)	9 (34.6)
Cardiovascular disease, N (%)	398 (40.16)	66 (30.3)	10 (19.2)	19 (39.6)	12 (46.2)
Neuropathy (MNSI$\geq$2), N (%)	656 (69.94)	128 (63.7)	27 (55.1)	27 (56.3)	20 (76.9)
Report ever smoking, N (%)	458 (47.46)	89 (41.6)	16 (31.4)	22 (45.8)	13 (50.0)
Report physical activity, N (%)	792 (78.26)	174 (80.9)	41 (78.9)	43 (89.6)	20 (76.9)
APOE (1 risk allele), N (%)	206 (26)	35 (20.0)	7 (16.7)	6 (15)	5 (19.2)
APOE (2 risk alleles), N (%)	9 (1.0)	1 (0.6)	0 (0)	1 (2.5)	1 (3.8)
Education (some college or higher), N (%)	925 (89.4)	204 (91.9)	44 (88.2)	33 (77.1)	21 (80.7)

Data are mean (± SD), median (lower-upper quartile) or number (percentage). Abbreviations eGFR: estimated glomerular filtration rate; ACR: albumin-to-creatinine ratio; SD: standard deviation; BMRI=brain MRI

Supplementary Table 2: Associations between clinical factors and cognitive function in T1D - multivariable analysis

	Unadjusted		Multivariable	
	Est (SE)	P	Est (SE)	P
**Psychomotor (D)**				
Age	4.62 (0.82)	<.0001	1.69 (1.17)	0.1527
Education	-61.95 (24.82)	0.0134	-51.12 (25.6)	**0.0477**
Duration	4.94 (0.92)	<.0001	3.75 (1.37)	**0.007**
eGFR	-0.69 (0.3)	0.0209	0 (0.34)	0.9893
ACR	8.03 (2.88)	0.0058	-2.19 (3.55)	0.5388
PDR	37.95 (13.11)	0.0042	20.96 (13.46)	0.1216
CRP	6.15 (3.12)	0.0498	-1.57 (3.33)	0.6379
Visual acuity	94.06 (13.42)	<.0001	87.59 (16.73)	**<.0001**
IL-6	34.09 (13.54)	0.0127	26.16 (11.72)	**0.0272**
**Psychomotor (ND)**				
Age	6.31 (1.11)	<.0001	1.88 (2.04)	0.3589
Education	-79.41 (34.49)	0.0223	-113.95 (44.19)	**0.0113**
Duration	8.06 (1.21)	<.0001	7.64 (2.55)	**0.0035**
SBP	0.99 (0.47)	0.0363	-1.32 (0.7)	0.0645
eGFR	-0.79 (0.4)	0.0497	0.5 (0.54)	0.3529
ACR	12.19 (3.9)	0.002	4.81 (5.96)	0.4212
PDR	34.05 (17.88)	0.0584	24.77 (22.96)	0.2831
CAC	7.36 (3.57)	0.0413	-0.43 (3.77)	0.9085
Visual acuity	111.58 (20.13)	<.0001	118.35 (28.83)	**<.0001**
IL-1β	19.62 (9.16)	0.0336	2.73 (9.11)	0.7652
IL-6	45.3 (18.31)	0.0143	32.22 (21.32)	0.1337
**Immedicate recall**				
Female	8.77 (1.93)	<.0001	8.6 (2.84)	**0.0031**
HDLc	0.22 (0.06)	<.0001	0.11 (0.12)	0.3697
Total cholesterol	0.09 (0.03)	0.0022	-0.04 (0.05)	0.353
TG-HDL ratio	-3.21 (1.39)	0.0217	-1.45 (2.36)	0.5406
eIS	6.87 (3.32)	0.0398	0.31 (5.01)	0.9506
CVD	-6 (1.96)	0.0025	0.46 (2.73)	0.867
CAC	-0.97 (0.42)	0.0217	-0.51 (0.42)	0.2327
**Delayed recall**				
Female	8.18 (2.07)	0.0001	6.72 (2.2)	**0.0026**
CRP	1.05 (0.53)	0.0476	0.64 (0.59)	0.2789
IL-1β	-2.27 (1.05)	0.0325	-2.45 (1.05)	**0.0208**
IFN-γ	2.27 (1.07)	0.0345	2.12 (1.05)	**0.0449**
**Executive function**				
Age	-0.07 (0.03)	0.0134	-0.02 (0.05)	0.6728
Duration	-0.08 (0.03)	0.0105	-0.08 (0.06)	0.184
LDLc	-0.02 (0.01)	0.0138	0.02 (0.02)	0.4961
Total cholesterol	-0.01 (0.01)	0.0165	-0.04 (0.02)	**0.0318**
Triglyceride	-0.92 (0.42)	0.0302	-1.04 (0.54)	0.0585
ACR	-0.21 (0.09)	0.0253	0 (0.12)	0.9812
CAC	-0.19 (0.09)	0.036	-0.08 (0.09)	0.373
Visual acuity	-1.4 (0.55)	0.0118	-0.95 (0.71)	0.1851
IL-1β	-0.43 (0.21)	0.0427	-0.5 (0.23)	**0.0306**
**Working memory**				
Education	0.37 (0.18)	0.0431	0.29 (0.18)	0.1126
Triglyceride	-1.5 (0.38)	0.0001	-1.17 (0.69)	0.0895
TG-HDL ratio	-0.86 (0.29)	0.0032	-0.41 (0.74)	0.5815
eIS	1.26 (0.62)	0.0427	0.01 (0.78)	0.9872
VAI	-0.01 (0)	0.0336	0 (0.01)	0.732
CVD	-0.78 (0.38)	0.0408	-0.41 (0.39)	0.2959
**Global cognition mis**				
Age at diagnosis	-0.13 (0.06)	0.0438	-0.09 (0.06)	0.0984
Female	1.71 (0.66)	0.0108	2.05 (0.61)	**0.0011**
SBP	-0.06 (0.02)	0.0096	-0.07 (0.02)	**0.0008**
eGFR	-0.04 (0.02)	0.0215	-0.04 (0.02)	**0.0452**
CML	1.16 (0.45)	0.012	0.8 (0.51)	0.1226
**Global cognition total**				
Age	-0.08 (0.04)	0.03	-0.05 (0.04)	0.2197
SBP	-0.03 (0.02)	0.0295	-0.01 (0.02)	0.6031
Visual acuity	-1.45 (0.6)	0.0173	-1.34 (0.63)	**0.0376**
CAC	-0.18 (0.09)	0.0444	-0.11 (0.09)	0.224

Est (SE): Estimate (standard error) from linear regression models tesing cognitive parameters as dependent variables and clinical parameters as independent.
Unadjusted analysis examines bivariate relationships between each clinical variable and cognitvie parameter, multivariable analysis examines all variables in same model. D, ND: Dominant and non-dominant hands; mis: Memory index score; eGFR: estimated glomerular filtration rate;
ACR: Urine albumin creatinine ratio; PDR: proliferative diabetic retinopathy; CVD: cardiovascular disease; CRP: C-reactive protein; SBP: systolic blood pressure
CAC: coronary artery calcification; TG: Triglyceride; eIS: estimated insulin sensitivity; VAI: visceral adiposity index; CML: carboxymethyl-lysine

Supplementary Table 3: Associations between physical or lifestyle activities and cognitive function

Activity	Cognitive Function	Estimate	SE	*p*
**Physical activity (Paffenbarger, N=150)**				
↑ Stairs climbed/day	↑ Executive function	0.074	0.03	0.014
↑ Blocks walked/day	↑ psychomotor speed & efficiency (ND)	0.857	0.378	0.025
↑ Kcal/week for swimming	↑ Delayed recall	0.011	0.005	0.043
↑ Duration/week for swimming	↑ Delayed recall	5.936	2.84	0.038
**Lifestyle activity, frequency/month (LAQ, N=62)**				
↑ driving or using public transportation	↑ executive function	0.08	0.03	**0.031**
↑ going to movies	↓ executive function	-0.96	0.45	**0.039**
↑ sewing	↓ psychomotor speed & efficiency (D)	-3.55	1.11	**0.003**
↑ sewing	↓ psychomotor speed & efficiency (ND)	-5.09	1.6	**0.003**
↑ talking about local/national issues	↓ psychomotor speed & efficiency (ND)	-2.62	1.27	**0.044**

Models adjusted for age, sex, and education. SE= Standard error.

Supplementary Table 4. Associations of dietary patterns with cognitive function (N=160)

Cognitive function	aMed		DASH		MIND		EDIH		EDIP	
	Est (SE)	*P*	*Est (SE)*	*P*	*Est (SE)*	*P*	*Est (SE)*	*P*	*Est (SE)*	*P*
Psychomotor function (D)	-2.96 (4.53)	0.514	0.36 (2.12)	0.864	-4.44 (3.39)	0.192	-6.08 (11)	0.581	6.25 (7.67)	0.416
Psychomotor function (ND)	-4.33 (5.68)	0.447	-0.27 (2.73)	0.920	-4.71 (4.82)	0.330	-6.08 (11)	0.581	3.78 (9.6)	0.694
Working memory	0.27 (0.11)	**0.019**	0.17 (0.05)	**0.002**	0.22 (0.12)	0.063	0.61 (0.22)	**0.007**	0.19 (0.2)	0.327
Delayed recall	-0.92 (0.74)	0.218	-0.56 (0.34)	0.105	0.67 (0.64)	0.299	-0.74 (1.45)	0.609	-0.22 (1.26)	0.865
Immediate recall	-0.69 (0.64)	0.281	-0.23 (0.31)	0.447	0.68 (0.6)	0.260	0 (1.25)	1.000	0.06 (1.09)	0.955
Executive function	0.06 (0.14)	0.651	0.04 (0.06)	0.496	-0.02 (0.13)	0.902	-0.44 (0.27)	0.100	-0.67 (0.23)	**0.003**
Global cognition (total)	0.16 (0.18)	0.368	0.04 (0.08)	0.644	0.07 (0.17)	0.684	-0.12 (0.36)	0.745	0.02 (0.29)	0.938
Global cognition (mis)	0.16 (0.26)	0.532	0 (0.11)	0.965	0.03 (0.27)	0.906	-0.77 (0.5)	0.130	0.08 (0.41)	0.853

aMed: alternate Mediterranean; DASH: Dietary Approaches to Stop Hypertension; MIND: Mediterranean-DASH Intervention for Neurodegenerative Delay;
EDIH: Empirical Index for Hyperinsulinemia; EDIP: Empirical Dietary Inflammatory Pattern; D: dominant hand; ND: non-dominant hand; mis: Memory index score.
Higher scores for all dietary indices represent higher adherence to (aMed, DASH, MIND) or higher intake (EDIH, EDIP) of healthier diets.
Estimates (Est), standard errors (SE) and p-values obtained from generalized linear regression models testing associations of dietary pattern to cognitive function.

Supplementary Table 5: Associations between brain volumes and cognitive function in T1D (n=52)

Cognitive parameter	Total brain		Total white matter		Total gray matter		Frontal lobe		Parietal lobe		Occipital lobe		Temporal lobe		Hippocampal		Alzheimer's disease signature region		Deep gray matter	
	Est (SE)	***P***	***Est (SE)***	***P***	***Est (SE)***	***P***	***Est (SE)***	***P***	***Est (SE)***	***P***	***Est (SE)***	***P***	***Est (SE)***	***P***	***Est (SE)***	***P***	***Est (SE)***	***P***	***Est (SE)***	***P***
**Immediate recall**																				
Model 1	-0.04 (0.03)	0.193	-0.07 (0.05)	0.168	-0.05 (0.05)	0.339	-0.17 (0.18)	0.352	-0.06 (0.26)	0.811	-0.89 (0.41)	**0.035**	-0.15 (0.26)	0.57	-0.67 (3.01)	0.826	-0.14 (0.44)	0.75	-0.29 (0.87)	0.744
Model 2	-0.02 (0.04)	0.709	-0.04 (0.06)	0.546	0.01 (0.08)	0.917	-0.13 (0.23)	0.586	0.13 (0.28)	0.637	-0.73 (0.59)	0.220	0.35 (0.4)	0.382	5.45 (3.83)	0.161	0.48 (0.53)	0.373	0.4 (0.97)	0.678
**Delayed recall**																				
Model 1	-0.01 (0.03)	0.686	-0.04 (0.05)	0.447	0 (0.05)	0.946	0.03 (0.18)	0.853	0 (0.25)	0.986	-0.6 (0.39)	0.134	0.07 (0.25)	0.767	1.3 (2.83)	0.647	0.1 (0.41)	0.812	-0.06 (0.82)	0.939
Model 2	-0.01 (0.04)	0.753	-0.04 (0.06)	0.471	0.03 (0.08)	0.714	0.01 (0.23)	0.980	0.06 (0.27)	0.829	-0.95 (0.57)	0.105	0.32 (0.39)	0.414	4.78 (3.79)	0.214	0.34 (0.53)	0.528	0.03 (0.96)	0.976
**Psychomotor (D)**																				
Model 1	-0.43 (0.07)	**3.00E-07**	-0.64 (0.14)	**2.00E-05**	-0.83 (0.14)	**6.00E-07**	-2.27 (0.57)	**2.00E-04**	-3.13 (0.75)	**1.00E-04**	-4.93 (1.28)	**4.00E-04**	-3.87 (0.69)	**1.00E-06**	-44.2 (8.0)	**1.00E-06**	-5.9 (1.2)	**1.00E-05**	-10.3 (2.5)	**1.00E-04**
Model 2	-0.32 (0.12)	**0.009**	-0.35 (0.17)	**0.050**	-0.6 (0.24)	**0.016**	-1.03 (0.73)	0.164	-1.92 (0.76)	**0.015**	-1.5 (1.79)	0.407	-2.61 (1.18)	**0.031**	-30.25 (10.85)	**0.008**	-3.29 (1.54)	**0.038**	-5.29 (2.68)	**0.055**
**Psychomotor (ND)**																				
Model 1	-0.78 (0.19)	**1.00E-04**	-1.2 (0.33)	**7.00E-04**	-1.48 (0.37)	**3.00E-04**	-3.5 (1.45)	**0.02**	-5.31 (1.86)	**0.007**	-5.8 (3.3)	0.086	-7.78 (1.73)	**5.00E-05**	-97.1 (19.4)	**9.00E-06**	-11.3 (3.0)	**6.00E-04**	-19.4 (6.1)	**0.003**
Model 2	-0.81 (0.3)	**0.011**	-0.88 (0.45)	**0.058**	-1.48 (0.6)	**0.018**	-1.94 (1.86)	0.305	-3.93 (1.97)	**0.052**	0.2 (4.6)	0.965	-8.98 (2.85)	**0.003**	-91.89 (26.86)	**0.001**	-8.79 (3.97)	**0.033**	-11.96 (6.86)	0.089
**Working memory**																				
Model 1	0 (0)	0.787	0 (0.01)	0.74	0 (0.01)	0.882	0 (0.03)	0.893	0 (0.04)	0.909	0.05 (0.06)	0.418	0.01 (0.04)	0.87	-0.32 (0.43)	0.471	0.03 (0.06)	0.647	-0.08 (0.13)	0.554
Model 2	0 (0.01)	0.818	0 (0.01)	0.862	-0.01 (0.01)	0.510	-0.04 (0.03)	0.205	-0.03 (0.04)	0.496	0.07 (0.08)	0.414	-0.03 (0.06)	0.635	-0.34 (0.56)	0.545	0.01 (0.08)	0.925	-0.09 (0.14)	0.536
**Executive function**																				
Model 1	0.01 (0.01)	**0.018**	0.03 (0.01)	**0.004**	0.02 (0.01)	0.176	0.03 (0.04)	0.381	0.03 (0.05)	0.578	0.14 (0.09)	0.114	0.09 (0.05)	0.11	1.32 (0.61)	**0.036**	0.13 (0.09)	0.147	0.05 (0.17)	0.762
Model 2	0.02 (0.01)	**0.027**	0.03 (0.01)	**0.007**	0.01 (0.02)	0.496	0.02 (0.05)	0.717	-0.01 (0.06)	0.805	0.13 (0.12)	0.322	0.1 (0.08)	0.265	1.68 (0.8)	**0.043**	0.09 (0.11)	0.441	-0.03 (0.19)	0.889
**Global (MIS)**																				
Model 1	0 (0)	0.506	0 (0.01)	0.691	-0.01 (0.01)	0.405	-0.03 (0.03)	0.362	-0.06 (0.04)	0.146	-0.11 (0.07)	0.122	-0.03 (0.04)	0.469	-0.3 (0.5)	0.554	-0.11 (0.07)	0.136	-0.13 (0.14)	0.381
Model 2	0 (0.01)	0.575	0.01 (0.01)	0.545	0 (0.01)	0.811	-0.01 (0.04)	0.873	-0.05 (0.05)	0.321	-0.07 (0.1)	0.507	0.03 (0.07)	0.647	0.28 (0.68)	0.681	-0.08 (0.1)	0.416	-0.05 (0.18)	0.788
**Global (Total)**																				
Model 1	0.01 (0)	0.096	0.01 (0.01)	0.124	0.01 (0.01)	0.125	0.02 (0.02)	0.38	0.02 (0.03)	0.414	0.03 (0.05)	0.539	0.06 (0.03)	0.059	0.62 (0.34)	0.074	0.07 (0.05)	0.146	0.01 (0.1)	0.908
Model 2	0.01 (0.01)	0.242	0.01 (0.01)	0.303	0.01 (0.01)	0.367	0.01 (0.03)	0.843	0 (0.03)	0.926	-0.02 (0.07)	0.788	0.08 (0.05)	0.108	0.78 (0.46)	0.100	0.05 (0.07)	0.452	-0.07 (0.11)	0.561

Model 1: Adjusted by Intracranial vol. (ICV) ; Model 2: adjustment by age, sex, education and ICV
Est: Estimate SE: Standard error ; D: dominant hand; ND: non-dominant hand; MIS: Memory index score.

Supplementary Table 6: Associations between clinical factors and brain volumes in T1D - multivariable analysis

	Unadjusted		Multivariable	
	**Est (SE)**	**P**	**Est (SE)**	**P**
**Total brain**				
Age	-6.282 (1.93)	0.002	-1.34 (1.77)	0.453
Education	45.542 (9.91)	3.20E-05	17.27 (27.98)	0.541
Duration	-6.542 (2.11)	0.003	-4.08 (1.84)	**0.033**
Female	-119.088 (21.73)	1.50E-06	-65.22 (20.45)	**0.003**
Waist-hip ratio	336.597 (121.75)	0.008	66.38 (86.53)	0.448
Exercise	70.009 (29.31)	0.021	14.32 (20.26)	0.484
Total cholesterol	-0.713 (0.31)	0.027	-0.19 (0.26)	0.459
HDLc	-2.008 (0.66)	0.004	-1.03 (0.57)	0.077
Visual acuity	-82.50 (29.46)	0.0073	-67.34 (21.16)	**0.003**
IL-1β	-57.57 (18.13)	0.0026	-32.06 (11.88)	**0.010**
**Total white matter**				
Age	-3.308 (1.14)	0.006	-0.37 (1.21)	0.764
Education	21.705 (6.21)	0.001	4.67 (19.46)	0.812
Duration	-3.493 (1.24)	0.007	-2.63 (1.29)	**0.049**
Female	-57.714 (13.73)	1.11E-04	-30.52 (13.44)	**0.029**
Exercise	37.137 (17.17)	0.035	9.85 (14.19)	0.492
Total cholesterol	-0.46 (0.18)	0.013	-0.29 (0.18)	0.104
HDLc	-1.005 (0.39)	0.014	-0.38 (0.4)	0.345
Visual acuity	-44.42 (17.14)	0.013	-38.9 (14.8)	**0.012**
IL-1β	-29.79 (10.74)	0.0079	-18.36 (8.25)	**0.032**
**Total gray matter**				
Age	-2.974 (1.03)	0.006	-0.61 (1.01)	0.552
Education	23.837 (5.15)	2.83E-05	13.42 (15.78)	0.401
Duration	-3.049 (1.12)	0.009	-1.4 (1.04)	0.188
Female	-61.374 (11.47)	2.29E-06	-33.83 (11.21)	**0.005**
Waist-hip ratio	196.36 (62.47)	0.003	52.09 (48.13)	0.287
Exercise	32.872 (15.5)	0.039	2.72 (11.37)	0.812
ACR	-5.53 (2.73)	0.049	-2.68 (1.95)	0.179
HDLc	-1.003 (0.35)	0.006	-0.52 (0.27)	0.068
Visual acuity	-38.08 (15.10)	0.015	-26.75 (11.86)	**0.031**
IL-1β	-27.78 (9.63)	0.006	-13.41 (6.72)	**0.054**
**Frontal lobe**				
Education	6.156 (1.54)	2.00E-04	10.54 (4.98)	**0.041**
Female	-14.228 (3.69)	3.00E-04	-8.49 (4.05)	**0.043**
HDL	-0.246 (0.1)	0.023	-0.13 (0.09)	0.168
PDR	-7.949 (3.65)	0.035	-4.52 (3.11)	0.155
IL-1β	-6.701 (2.89)	0.025	-2.31 (2.43)	0.349
**Parietal lobe**				
Age	-0.493 (0.22)	0.031	0.11 (0.28)	0.698
Education	2.768 (1.23)	0.029	3.19 (4.14)	0.446
Duration	-0.681 (0.23)	0.005	-0.38 (0.29)	0.187
Female	-6.003 (2.89)	0.043	-2.8 (2.92)	0.345
Waist-hip ratio	43.788 (12.87)	0.001	22.55 (14.55)	0.129
ACR	1.39 (0.57)	0.018	-1.19 (0.58)	**0.045**
TNF-α	-6.18 (2.83)	0.03	-2.97 (2.73)	0.282
**Occipital lobe**				
Female	-7.86 (1.45)	1.80E-06	-5.2 (1.47)	**0.001**
Age	-0.364 (0.13)	0.008	-0.28 (0.1)	**0.007**
Education	2.368 (0.71)	0.002	2.22 (2.14)	0.306
Total cholesterol	-0.06 (0.02)	0.01	-0.02 (0.02)	0.230
HDLc	-0.133 (0.04)	0.004	-0.03 (0.04)	0.532
Visual acuity	-4.06 (1.97)	0.045	-2.18 (1.56)	0.169
IL-1β	-2.89 (1.26)	0.026	-1.12 (0.89)	0.216
**Temporal lobe**				
Age	-0.635 (0.21)	0.004	-0.22 (0.21)	0.302
Education	4.949 (1.07)	2.70E-05	4.16 (3.29)	0.214
Duration	-0.651 (0.23)	0.007	-0.32 (0.22)	0.147
Female	-13.105 (2.32)	7.80E-07	-8.31 (2.35)	**0.001**
Waist-hip ratio	38.407 (13.01)	0.005	6.2 (10.01)	0.540
Exercise	6.966 (3.19)	0.034	1.16 (2.39)	0.628
HDLc	-0.198 (0.07)	0.009	-0.09 (0.06)	0.124
Visual acuity	-7.65 (3.17)	0.02	-4.94 (2.49)	**0.055**
IL-1β	-5.37 (2.0)	0.01	-2.26 (1.4)	0.115
**Hippocampal**				
Age	-0.06 (0.02)	0.003	-0.01 (0.02)	0.736
Education	0.33 (0.1)	0.002	0.14 (0.31)	0.648
Duration	-0.06 (0.02)	0.003	-0.03 (0.02)	0.130
Female	-1.07 (0.21)	6.30E-06	-0.77 (0.23)	**0.002**
Waist-hip ratio	3.215 (1.15)	0.007	0.91 (1.01)	0.373
ACR	-0.11 (0.05)	0.027	-0.08 (0.04)	0.059
HDLc	-0.02 (0.01)	0.02	-0.01 (0.01)	0.321
IL-1β	-0.46 (0.17)	0.01	-0.29 (0.14)	**0.041**
**Alzheimer's ds sig region**				
Age	-0.348 (0.13)	0.01	0.14 (0.13)	0.285
Education	2.389 (0.69)	0.001	-0.66 (2.03)	0.748
Duration	-0.435 (0.14)	0.003	-0.36 (0.14)	**0.012**
Female	-6.293 (1.56)	2.00E-04	-3.16 (1.43)	**0.034**
Waist-hip ratio	25.703 (7.73)	0.002	6.69 (6.56)	0.315
BMI	0.334 (0.15)	0.029	0.12 (0.12)	0.294
Exercise	4.313 (1.93)	0.03	0.65 (1.5)	0.668
HDLc	-0.104 (0.04)	0.024	-0.08 (0.04)	**0.028**
ACR	-0.79 (0.34)	0.027	-0.43 (0.25)	0.102
Visual acuity	-4.76 (1.91)	0.017	-4.81 (1.51)	**0.003**
IL-1β	-3.09 (1.22)	0.015	-1.97 (0.91)	**0.037**
TNF-α	-3.53 (1.70)	0.042	-2.96 (1.23)	**0.022**
**Deep gray matter**				
Age	-0.153 (0.07)	0.025	-0.04 (0.09)	0.681
Education	1.07 (0.36)	0.005	-0.3 (1.19)	0.805
Duration	-0.181 (0.07)	0.014	-0.12 (0.1)	0.214
Female	-2.737 (0.81)	0.001	-2.4 (0.9)	**0.012**
Waist-hip ratio	13.04 (3.85)	0.001	6.17 (4.59)	0.188
Lifetime hypoglycemia severity	-0.087 (0.04)	0.026	-0.08 (0.03)	**0.019**
eIS	-2.665 (1.31)	0.047	-1.47 (1.35)	0.285

Est (SE): Estimate (standard error) from linear regression models tesing cognitive parameters as dependent variables and clinical parameters as independent.
Unadjusted analysis examines bivariate relationships between each clinical variable and cognitvie parameter, multivariable analysis examines all variables in same model. ACR: Albumin-creatinine ratio

Supplementary Table 7: Clinical history and brain pathology in 26 brain donors

				Clinical hx						Complications				Brain histopathology								
														Vascular					*Alzheimer's related*			
NO	Sex	Age	APOE	smoke	exercise	COD	Time	HbA1c (%)	HDL (mg/dL)	CVD	DN	DPN	PDR	Infarct	Hemo-rrhage	Arterio-sclerosis	Athero-sclerosis	CAA	Amyloid plaques	Braak Stage	Atrophy	Brain wt (g)
1	M	75	e3e3	Y	Y	U	1y	10.5	51	yes	no	.	.	yes	no	mild-mod	mod	no	+++	III-IV	mild-mod	1080
2	M	80	e3e4	Y	Y	MOD	6y	7.4	62	yes	no	.	Yes	no	no	mild-mod	mild	no	+	II	no	1270
3	F	77	e3e3	N	N	CVD	5y	6.2	51	yes	yes	.	.	no	no	mild-mod	no	no	negative	II	mild	1240
4	F	90	e3e4	N	Y	Ren.F	9y	7.5	100	yes	yes	Yes	Yes	no	no	mild-mod	mod	mild	++	II	mild	1005
5	F	82	e3e3	N	Y	CVD	8y	7.4	99	yes	no	Yes	no	no	no	no	mild	no	negative	NA	yes	900
6	M	80	e3e3	Y	N	Diab.	7y	7.8	43	yes	Yes	Yes	no	no	no	mild-mod	mild	no	negative	II;	mild	1110
7	M	80	e3e3	Y	Y	U	5y	6.8	66	no	No	yes	yes	yes	yes	mild-mod	mod	no	negative	I	no	1180
8	M	83	e4e4	Y	N	Resp.F	7y	7.5	76	yes	No	.	yes	no	no	mod	mild	mild-mod	+++	IV	mild	1140
9	M	81	e3e3	N	Y	U	7y	7.1	58	.	No	.	no	no	no	mild	no	no	++	III	no	1160
10	M	92	e3e3	Y	N	CVD	14y	6.4	38	no	No	no	no	no	no	mod-sev	no	no	NA	III	mild	1170
11	M	67	e3e3	Y	Y	Resp.F	15y	8	43	yes	No	yes	yes	yes	mvl	mild-mod	no	no	NA	III	no	1250
12	M	89	e3e4	Y	Y	AlzD.	14y	7.5	80	no	Yes	no	no	yes	no	mod	mild	mod	++	I-II	mild	1100
13	M	81	e2e3	N	Y	CVD	2y	5.1	51	yes	Yes	yes	.	yes	mvl	mod-sev	mild	no	NA	I	mild	1180
14	M	92	e2e3	Y	Y	CVD	2y	6.2	70	yes	No	.	yes	no	no	mod	mod	mild	+	III	mild	1080
15	M	86	e3e3	Y	N	Ren.F	0.6y	7	65	no	Yes	Yes	.	no	no	mod	mild	no	negative	I	no	1330
16	M	91	e2e3	N	Y	CVD	10y	7.2	52	no	No	.	no	no	yes	mild	mild	mod	negative	I	no	1320
17	M	80	e3e3	Y	Y	CVD	13y	6.8	55	yes	no	Yes	no	yes	no	mild-mod	mild	mild	+++	IV-V	mild	1250
18	M	70	e3e4	N	Y	CVD	9y	7.6	48	yes	no	.	no	no	no	mod	mild	mod	+	I	mild	1200
19	M	84	e3e3	N	Y	Acci	6y	6.7	36	no	No	.	yes	no	no	mild-mod	mild	no	++	III-IV	mild	1150
20	F	92	e3e3	N	Y	Cancer	12y	8.2	58	no	No	Yes	no	no	mvl	mild-mod	mild	no	+	III	mild	980
21	F	69	e3e3	N	N	CVD	13y	8.7	48	no	No	Yes	.	no	no	mild-mod	mild	no	negative	0	mild	1200
22	F	85	e3e3	N	Y	AlzD	16y	8.5	89	yes	No	Yes	.	no	no	mild-mod	mild	mild-mod	+++	V	mild	1010
23	M	71	-	Y	Y	Acc.	7y	7.2	90	yes	No	.	yes	no	no	mod	no	mod	++	III	no	1490
24	M	91	e2e3	-	Y	ESKD	16y	6.1	73	yes	Yes	.	Yes	No	mvl	mild-mod	No	mod	++	III	mild	1050
25	F	76	e3e3	Y	Y	CVD	11y	7.9	54	yes	No	Yes	no	no	no	mild-mod	no	mild-mod	negative	II	Mild	1260
26	M	80	e3e4	N	Y	CVD	5y	6.5	34	yes	No	.	no	no	no	mild-mod	mod	mild-mod	negative	I	mild	1390

COD: Cause of death; U: Unknown/Other; MOD: Multi-organ dysfunction; CVD: cardiovascular disease; Resp.F: Respiratory failure; AlzD: Alzheimer's disease; Ren.F: Renal failure;
Acci: Accident; ESKD: End-stage kidney disease; HDL: High-density lipoprotein; DN: diabetic nephropathy; DPN: diabetic peripheral neuropathy; PDR: Proliferative diabetic nephropathy.
Time: Time between last clinical visit and death
mod: moderate; sev: severe; mvl: micro-vascular lesions; Amyloid plaques: +++ frequent; ++ moderate; + infrequent; NA- not assessed.
Pt.17- family history of Alzheimer's; Pt 24<high school diploma; High school only – Pt 1,5,18; No HTN – 7,13,22,25; frequent hypoglycemia – Pt.7;
BMI>30 – Pt. 6,18; BMI>25: 8,10, 13,16, 19, 21,23; All patients LDL <160; Cholesterol: High (>240) in Pt. 1; borderline high (>200)- Pt 4,5; rest all had normal cholesterol at last visit.;
Triglycerides: High (>200) – Pt.1; borderline high (150-199) – Pt.6, 21; rest all had normal triglycerides at last visit

Supplementary Table 8: Longitudinal study (n=48) - associations between cognitive decline and visual acuity

Cognitive function	Average cognitive decline over follow-up	Association of cognitive change over follow-up with visual acuity at first cognitive visit	
	**Mean (SD)**	**Est (SE)**	**P**
**Psychomotor (D)**	-4.81 (40.4)	12.67 (16.6)	0.45
**Psychomotor (ND)**	-19.83 (100.6)	-217.15 (38.5)	**1.50E-06**
**Working memory**	0.19 (3.3)	-2.14 (1.5)	0.17
**Delayed recall**	-4.35 (17.8)	-6.03 (8.8)	0.5
**Immediate recall**	-1.98 (10.1)	-2.19 (5.1)	0.67
**Executive function**	0.54 (3.0)	1.9 (1.4)	0.19

Supplementary Table 9. Brain MRI Protocol as per the Alzheimer's disease Neuroimaging Initiative (ADNI)-3 advanced imaging protocol

Sequence Name	Approx. MRI time (min)	Outcomes
**MP-RAGE**	6:20	T1-weighted structure analysis including volumetric measures of whole brain and regional structures (hippocampus and entorhinal cortex), cortical thickness and atrophy. Analysis by FreeSurfer v.6 neuroimaging tool.
**3D FLAIR**	5:30	White matter disease, infarction, pathology, multi-spectral tissue segmentation
**High Res Hippo**	4:20	Hippocampal subfield measurement
**T2* GRE (T2*-weighted gradient echo)**	4:10	Cerebral microbleed assessment
**ASL**	4:00	Grey matter and white matter fluid maps, Cerebral blood flow
**Diffusion Tensor Imaging (DTI)**	7:10	Fractional anisotropy, apparent diffusion coefficient, mean diffusivity of white matter tracts. Analysis by DTIStudio, ROIEditor, and Diffeomap.
**EPI-BOLD**	10:00	Resting state fMRI analysis

Supplementary Table 10. Brain MRI volumes - description of Regions of Interest (ROIs)

ROI	Description
Total brain	Volume of total brain excluding ventricles
Total gray matter	Volume of all gray matter
Total white matter	Volume of total gray matter subtracted from total brain volumes excluding ventricles
Frontal lobe	Superior Frontal, Rostral and Caudal Middle Frontal, Pars Opercularis, Pars Triangularis, and Pars Orbitalis; Lateral and Medial Orbitofrontal;<br>Precentral; Paracentral; Frontal Pole
Parietal lobe	Superior Parietal; Inferior Parietal; Supramarginal; Postcentral; Precuneus
Occipital lobe	Lateral Occipital; Lingual; Cuneus; Pericalcarine
Temporal lobe	Superior, Middle, and Inferior Temporal;<br>Banks of the Superior Temporal Sulcus;<br>Fusiform; Transverse Temporal; Entorhinal; Temporal Pole; Parahippocampal
Hippocampal	
Alzheimer disease signature region	hippocampus + parahippocampal + entorhinal + inferior parietal lobule + precuneus + cuneus
Deep gray matter	thalamus + putamen + caudate + globus pallidus

Supplementary Fig.1. White matter hyperintensities (WMH) in T1D Medalists

A. WMH associations with cognitive function in T1D

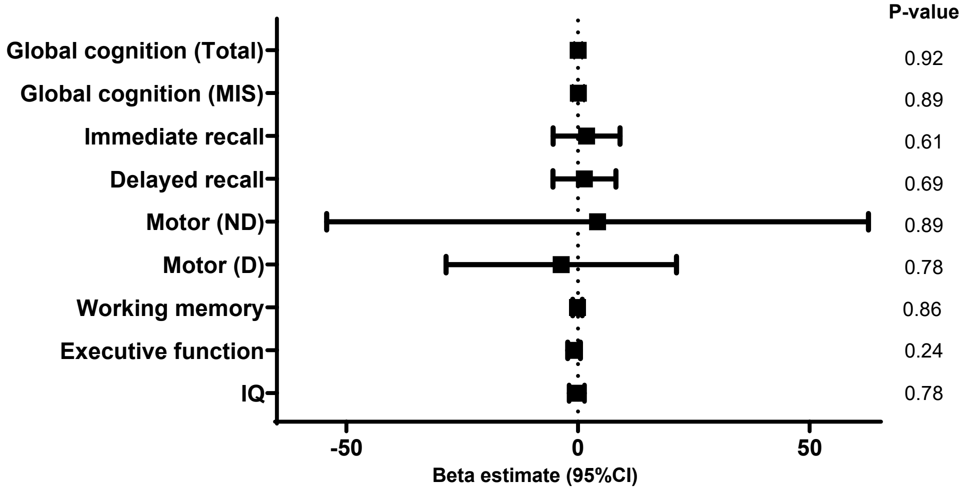

B. WMH associations with AGEs & kidney function in T1D

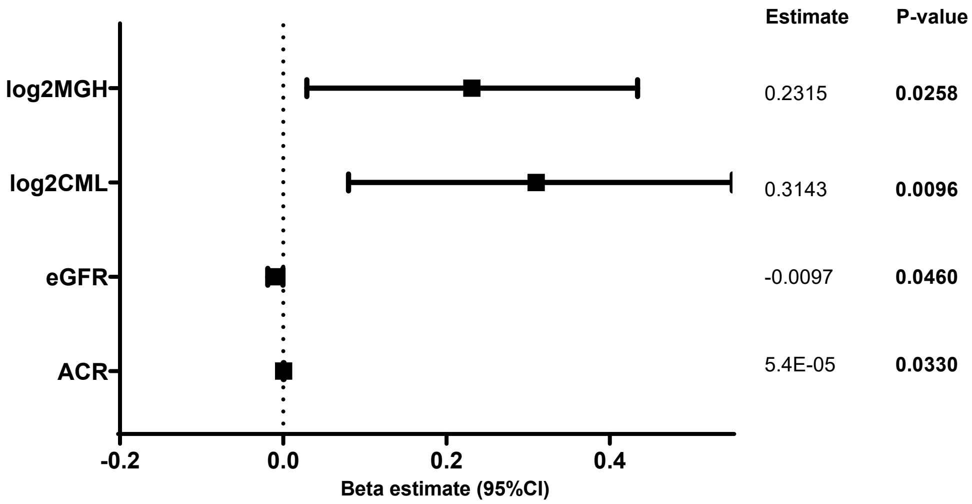

C. WMH associations with subclinical atherosclerosis in T1D

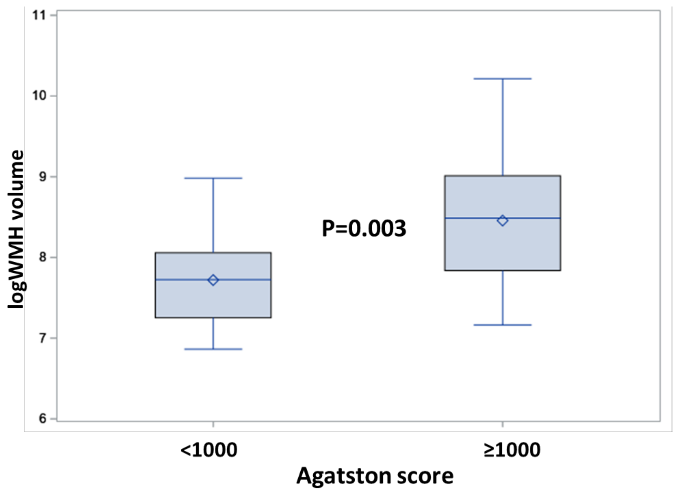

Supplementary Fig.2A-C. Cerebral Perfusion (ASL)

A.

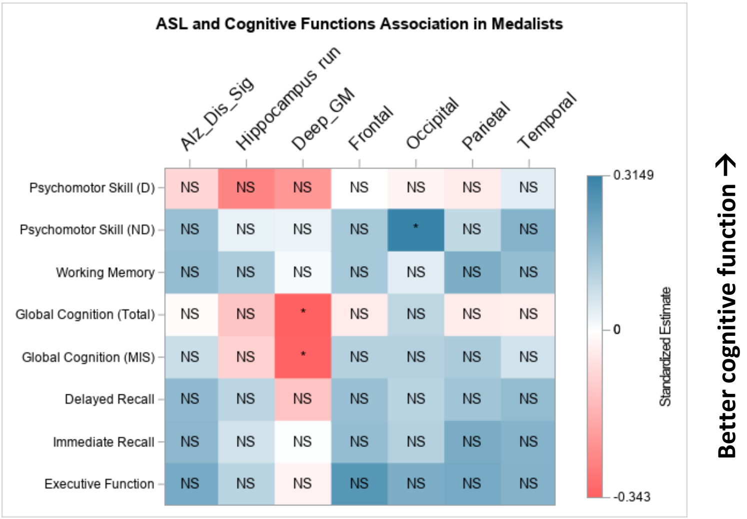

B.

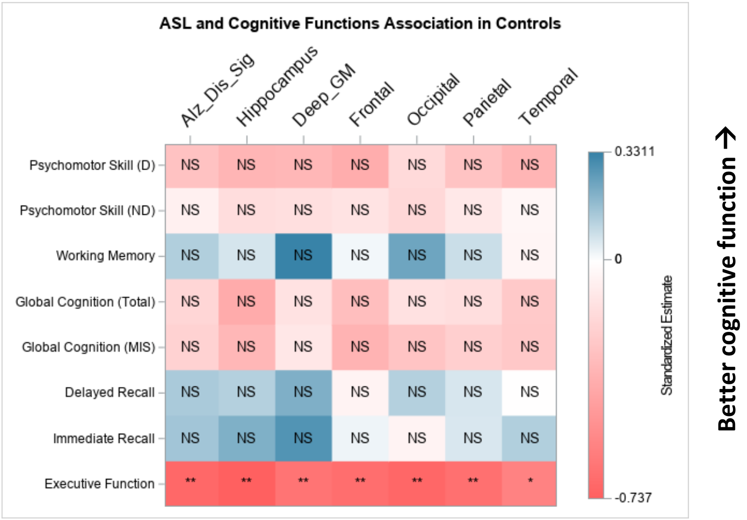

C. ASL And Clinical Characteristics In T1D Medalists

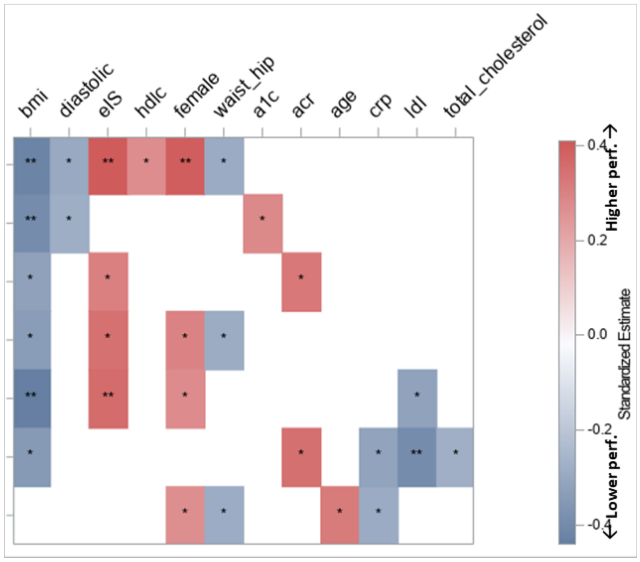

Remain p<0.05 in Multivariable models

BMI, sex

BMI

ACR

None

BMI, sex, LDL

ACR, LDL

Sex, Age, CRP

***p<0.0001
**p<0.01
*p<0.05

Supplementary Fig.3. Thickness of retinal layers (OCT-derived) and brain volumes

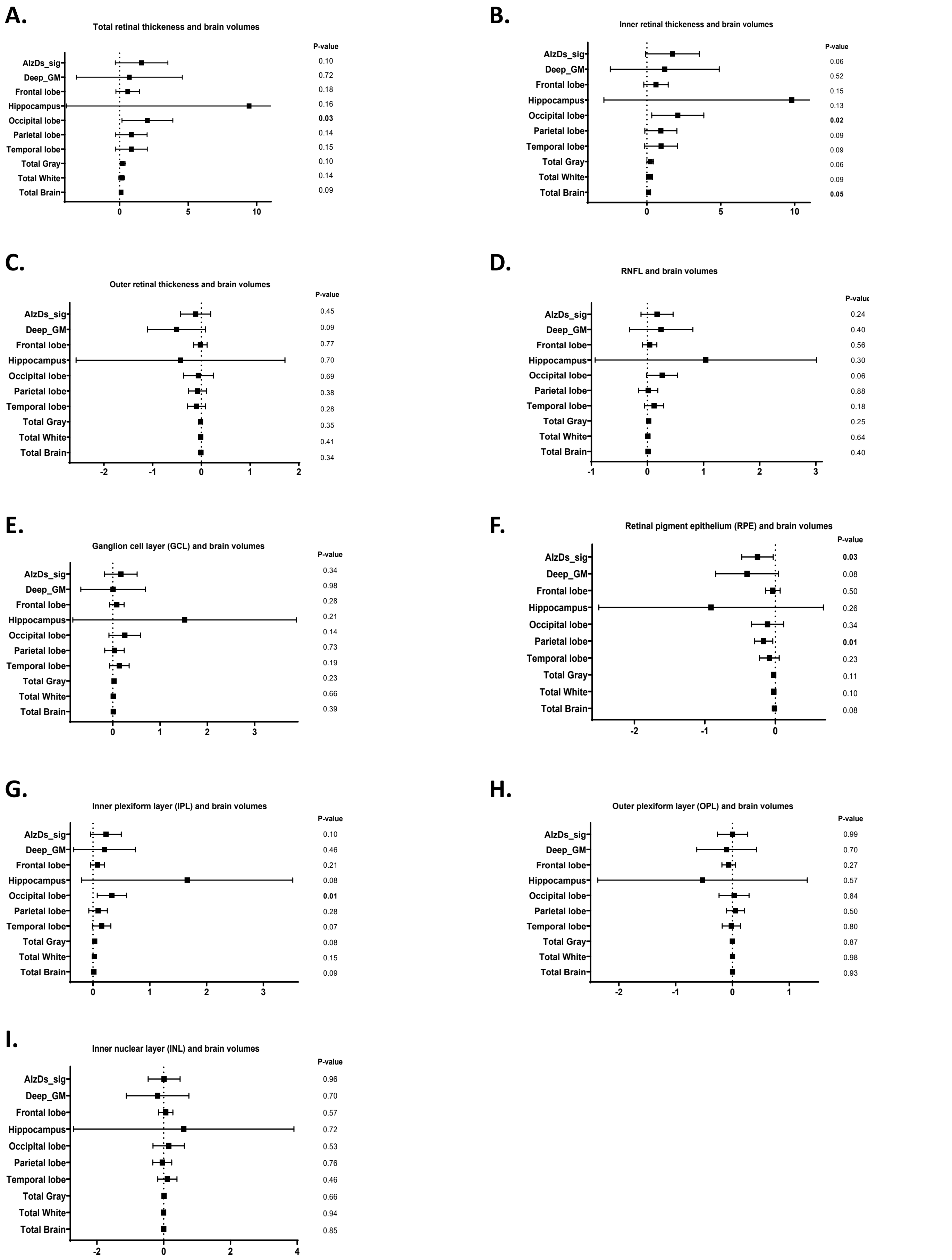

Supplementary Fig.4: Study design flowchart

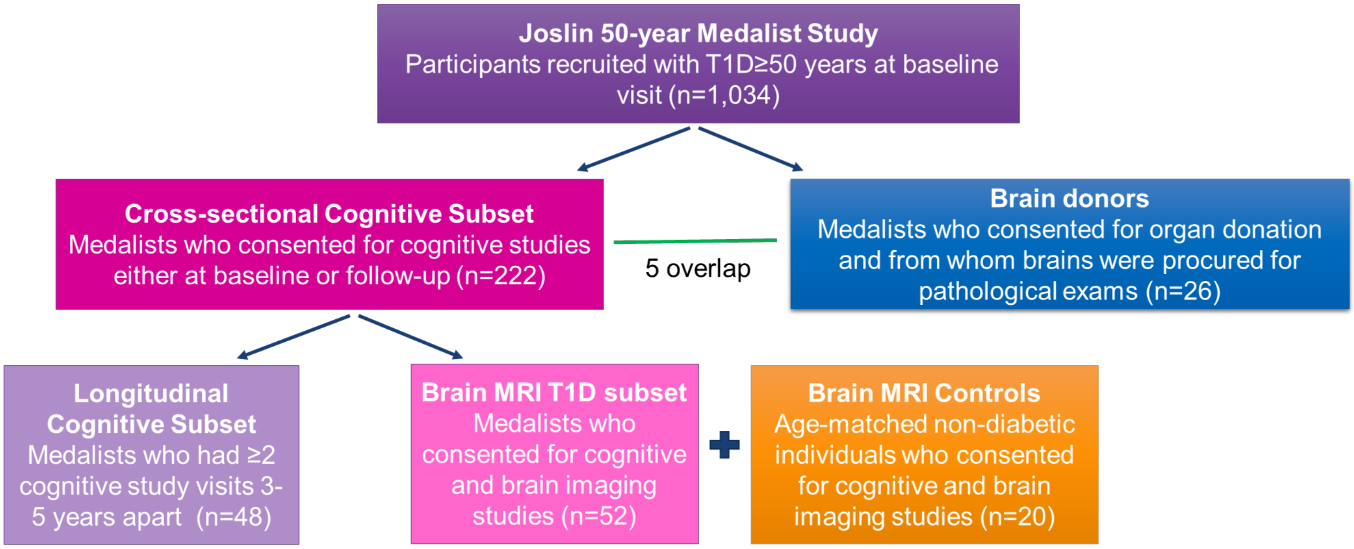

Supplement: Supplemental data [file jciinsight-10-180226-s096.pdf]
